# The effect of soil type on yield and micronutrient content of pasture species

**DOI:** 10.1371/journal.pone.0277091

**Published:** 2022-11-02

**Authors:** Tegan Darch, Martin S. A. Blackwell, Jessica Hood, Michael R. F. Lee, Jonathan Storkey, Deborah A. Beaumont, Steve P. McGrath

**Affiliations:** 1 Intelligent Data Ecosystems, Rothamsted Research, Okehampton, Devon, United Kingdom; 2 Net Zero and Resilient Farming, Rothamsted Research, Okehampton, Devon, United Kingdom; 3 Intelligent Data Ecosystems, Rothamsted Research, Harpenden, Hertfordshire, United Kingdom; 4 Office of the Deputy Vice-Chancellor, Harper Adams University, Newport, Shropshire, United Kingdom; 5 Protecting Crops and the Environment, Rothamsted Research, Harpenden, Hertfordshire, United Kingdom; 6 Sustainable Soils and Crops, Rothamsted Research, Harpenden, Hertfordshire, United Kingdom; University of Minnesota, UNITED STATES

## Abstract

The use of multispecies swards on livestock farms is growing due to the wide range of benefits they bring, such as improved biomass yield and animal performance. Preferential uptake of micronutrients by some plant species means the inclusion of legumes and forbs in grass-dominated pasture swards could improve micronutrient provision to livestock via careful species selection. However, although soil properties affect plant micronutrient concentrations, it is unknown whether choosing ‘best-performing’ species, in terms of their micronutrient content, needs to be soil-specific or whether the recommendations can be more generic. To address this question, we carried out an experiment with 15 common grass, forb and legume species grown on four soils for five weeks in a controlled environment. The soils were chosen to have contrasting properties such as texture, organic matter content and micronutrient concentrations. The effect of soil pH was tested on two soils (pH 5.4 and 7.4) chosen to minimise other confounding variables. Yield was significantly affected by soil properties and there was a significant interaction with botanical group but not species within a botanical group (grass, forb or legume). There were differences between botanical groups and between species in both their micronutrient concentrations and total uptake. Micronutrient herbage concentrations often, but not always, reflected soil micronutrient concentrations. There were soil-botanical group interactions for micronutrient concentration and uptake by plants, but the interaction between plant species (within a botanical group) and soil was significant only for forbs, and predominantly occurred when considering micronutrient uptake rather than concentration. Generally, plants had higher yields and micronutrient contents at pH 5.4 than 7.4. Forbs tended to have higher concentrations of micronutrients than other botanical groups and the effect of soil on micronutrient uptake was only significant for forbs.

## Introduction

Sustainable ruminant livestock production should minimise the use of potentially human-edible feeds like grain and be predominantly or entirely pasture or agri-food by-product based [1]. However, pasture-based livestock diets can be deficient in key micronutrients [2]. Although supplementation of the diet with minerals is possible, this is an added cost and with the use of prophylactic administration, which is common, can lead to toxicity through over-supplementation due to manufacturers formulating products to maximum permitted levels rather than livestock requirements. Furthermore, over supplementation of micronutrients has environmental risks via excretion with potential run-off to water courses [3]. Grazing of pastures that include legume and forb species, which tend to contain higher concentrations of micronutrients than grasses [4], may prevent micronutrient deficiencies in a more sustainable way. Forb and legume species are prevalent in multispecies swards, benefits of which include decreased N fertilizer requirements relative to monoculture grass or grass-clover systems [5]. There is increasing implementation of multispecies swards, and in the UK farmers can receive payments for implementing them through the Countryside Stewardship Scheme [6]. Consequently, there is the potential to improve sward micronutrient concentrations by careful selection of species into the sward. However, although we have some knowledge about how pasture species compare in their micronutrient contents, it is less clear how site-specific this might be, for example due to variable soil properties.

In a previous study we gathered empirical data on the micronutrient concentration of a wide range of species typically considered for inclusion in a multispecies sward in the UK [7]. The data indicated that inclusion of the forbs *Achillea millefolium*, *Cichorium intybus* and *Plantago lanceolata*, and the legumes *Medicago lupulina*, *Trifolium hybridum* and *Lotus corniculatus*, is likely to improve the Co, Cu, I, Se and Zn concentrations in the sward. These conclusions were based on growth on a single growth medium, but it is known that there are many soil factors which affect micronutrient uptake by plants [4]. In addition to soils varying in their total micronutrient concentration, it is known that soil properties such as pH, redox status, organic matter, texture and microbial activity all affect micronutrient availability to plants [4].

Therefore, we might expect the micronutrient concentration of a given pasture species to vary according to the soil in which it grows. Therefore, to select species that improve the micronutrient content of a multispecies sward, we need to understand whether the soil, and its effect on yield, also affects the relative ranking of species. There are few published studies where the relative ranking of pasture species across soils has been tested, and those that are available have some limitations that mean further data collection is warranted. For example, because the number of soils tested was low [e.g. 2 soils, 8], or the number of species tested was small [e.g. 8 perennial ryegrass varieties, or 2 grasses, 2 legumes and 1 forb, 9, 10]. In field studies, climatic differences will confound soil effects making it more challenging to interpret the data [10].

The effect of plant species and of soil type on plant micronutrient uptake, which we define as above ground dry matter yield multiplied by micronutrient concentration, will vary across micronutrients. Plants move nutrients into their cells via transporters, and there are often different transporters required for different micronutrients, although, for example, sulphate transporters can facilitate Mo and Se uptake [11]. Consequently, it does not follow that a plant species with a high uptake of one micronutrient will also show high uptake of other micronutrients. Micronutrients differ in their chemical form in which they are taken up by plants, including as divalent cations (Zn^2+^, Fe^2+^, Cu^2+^, Mn^2+^), and divalent anions (Mo as molybdate, MoO_4_^2-^; Se as selenate, SeO^2-^) [11]. As a result, pH can be a strong controlling factor on micronutrient availability, with cation availability greater at low pH and anion availability greater at high pH [12, 13]. Soil pH may therefore have a considerable influence on the relative ranking of plant species for their micronutrient content. Soil liming is common practice on farms and therefore understanding how pH affects the ranking of plant species for various micronutrients will also aid an understanding of how pasture management can affect sward micronutrient contents.

The aim of this study was to determine how soil type affects the relative ranking of pasture species for micronutrient content (concentration and total uptake) to inform choices about species selection in multispecies swards. We also aimed to determine whether there was a difference in our conclusions depending on which metric, concentration or uptake, was considered. These aims were addressed via a pot study in a controlled environment facility to reduce the confounding effects of climate and pasture management, using four soils chosen to have varying soil properties. Two of the soils were taken from the same field site but from areas where different management has resulted in a large pH difference to build up over time, enabling the effect of soil pH to be investigated while minimising other soil confounding variables. We hypothesised that the relative ranking of species for their micronutrient content would remain consistent across soil types, despite expected differences between species and between soil types. We therefore hypothesised that although micronutrient concentrations in pasture species are affected by soil type, there are species that can be consistently recommended to be ‘better performers’ and warrant inclusion in multispecies swards due to their micronutrient content.

## Materials and methods

## Soil and plant treatments

The plant treatment consisted of 15 pasture species from three botanical groups, namely grasses, legumes and forbs (supplied by Germinal Ltd, Linconshire, UK, except *P*. *lanceolata*, supplied by Cotswold Seeds, Moreton-in-Marsh, UK). Grass species were *Lolium multiflorum* cv. Fox, *Phleum pratense* cv. Comer, *Anthoxanthum odoratum*, *Dactylis glomerata* cv. Amba, *Holcus lanatus*, and *Lolium perenne* cv. Abergain. Legume species were *Medicago Lupulina* cv. Virgo, *Trifolium Hybridum* cv. Ermo, *Trifolium pratense* cv. Merviot, *Trifolium repens* cv. Aberswan and *Lotus Corniculatus* cv. Leo. Forb species were *Cichorium Intybus* cv. Puna II, *Plantago Lanceolata* cv. Endurance, *Achillea millefolium* and *Sanguisorba minor*. Species were selected on the basis of being commonly included in multispecies sward mixtures and presenting a range of micronutrient concentrations in an earlier trial [7].

The soils Arable High pH (AH) and Arable Low pH (AL) were selected to investigate the effect of soil pH on micronutrient concentrations while minimising confounding variables. They were taken from a long-term experiment at Rothamsted Research (Hertfordshire, UK) known as the ‘Acid Strip’, where a pH gradient of 3.7 to 7.8 has developed due to differential application of lime in the 19^th^ century [14]. The soil is classified as a well-drained to moderately well-drained Typic Paleudalf with a flinty silty clay loam topsoil and is sown to wheat every year. Soils were taken from two locations along this strip, aiming for moderately acidic (pH 5.5) and moderately alkaline (pH 7.5) soils. At each location, soils were sampled down to 23 cm in a W across the width of the acid strip. Soils were collected in August 2020 following the wheat harvest. The pH of the AH soil (pH 7.3) was slightly lower than we were aiming for, and an acid-drop test indicated that the free carbonate, which might be responsible for the binding of micronutrients, was low. Therefore, we added additional calcium carbonate (CaCO_3_) to the AH soil. Preliminary tests indicated that a 0.03% addition of CaCO_3_ by weight increased the soil pH by ~0.2 units to pH 7.5 within 2 weeks when the soil was held at its water holding capacity, and that soil pH then stabilised. A grassland soil (NW) was sampled from the North Wyke Farm (Devon, UK). The soil was classified as an Aeric haplaquept, and a clayey typical non-calcareous pelosol in head from clay soil and had been under permanent pasture with a history of minimal fertilizer application. Soils were collected from the loose topsoil of land recently ploughed to 15cm, in a W across the field. Finally, a growing medium (GM) was used, which contained 80% sterilised loam, 15% 2EW sand and 5% lime free grit of < 5mm (‘Rothamsted prescription soil’, Petersfield Growing Mediums, Leicester, UK). As well as providing an additional combination of nutrient concentrations, textural properties, pH and soil organic matter on which to test the hypotheses, the GM also provided continuity with the study of Darch, McGrath [7]. In both studies, the GM was used to grow the same species but with different growing conditions and plant maturities at harvest, enabling an investigation of the effect of growing conditions on plant nutrient concentrations, although that is beyond the scope of this paper. Although technically a growing medium rather than a soil, GM is referred to as a soil throughout this manuscript for brevity. All soils were air-dried and sieved to <10 mm before use.

Three replicates of 200 g dry weight of each soil type, after addition of CaCO_3_ to the AH soil, were maintained at 60% water holding capacity (WHC) for two weeks using milli-Q (ultra-high pure) water. Soils were kept in a controlled environment room which was maintained at 21/16 degrees at day/night, with a 16/8 hour cycle. After four weeks, moist soil was analysed for nitrate and ammonium concentrations using a potassium chloride (KCl) extraction (2:1 KCl:soil) with 1 hour shaking and filtration through Whatman grade 2 filter papers (8 μm particle retention). Total oxidised N (TON) and ammonium-N (NH_4_-N) were analysed using an Aquachem 250 discrete photometric analyser (Thermo Fisher Scientific). The remainder of the soil was air-dried and sieved to <2mm and analysed for Olsen extractable P [15] and ammonium nitrate extractable K and Mg [16] in the laboratories of NRM (Berkshire, UK). Based on these data [17], rates of NPK addition were determined according to the RB209 recommendations for farmers in the UK [18], which give NPK application levels according to the NPK concentrations in the soil and are assumed to bring them up to an optimal NPK level. These optimal levels were 16–25 mg/l Olsen extractable P and 121–180 mg/l Ammonium nitrate extractable K, and we followed the recommendation that N should only be applied to soils with a N concentration of less than 50 kg N/ha (~13 mg/kg soil) when leguminous plants are to be sown [17]. Application levels were: 13.87, 22.19, 22.19 and 0 mg P/pot for NW, AH, AL and GM soils respectively, as NaH_2_PO_4_.6H_2_O; 32, 16, 16 and 16 mg K/pot for NW, AH, AL and GM soils respectively, as KCl, and 0, 32, 32 and 0 mg N/pot for NW, AH, AL and GM soils respectively, as ammonium nitrate.

To assess soil nutrient concentrations and pH after NPK addition, ultrapure (MQ) water was applied to each soil type and maintained at 60% WHC for two weeks as before. The NPK were applied to the soil surface in a dilute form to ensure even distribution through the soils and left for a further four days. The available NPK and Mg were analysed again as previously described. In addition, soil pH was measured in water (1:2.5 soil:water), and Mehlich III extractable Cu, Zn, Fe, S, Co and Mn were analysed by Inductively Coupled Plasma–Optical Emission Spectrometer (ICP-OES, NRM, Berkshire, UK) [19]. Total micronutrient concentrations were determined via aqua regia digest for soil [20] and nitric perchloric digestion for plant material [21], followed by Inductively Coupled Plasma–Mass Spectrometer analysis (ICP-MS, NexION 300X, Perkin Elmer) and ICP-OES analysis (Optima 7300 DV, Perkin Elmer). Soil organic carbon (SOC) was measured by loss on ignition (450°C for 10 hours). All analyses were conducted in triplicate.

### Plant growth trial

The experiment was a full factorial pot experiment with 4 replicates of each soil and plant species combination. The experimental layout was a randomised complete block design with latinized sub-blocking with 3 equal sized subblocks per replicate, to account for potential edge effects caused by greater air movement at the side of the room leading to greater transpiration. Randomisation was carried out using CycDesigN (VSNi) with treatment grouping defined to ensure an even distribution of species from the same botanical group and grown in the same soil across the layout. The growth trial took place in controlled environment facilities with the same day/night conditions as previously described.

Cylindrical pots of 9 cm diameter and 30 cm height were loosely filled with soil after preparation as described above. Soils were wetted to 60% WHC using Milli-Q water and maintained at this moisture content for two weeks, covered with black plastic to minimise water loss. Seeds were then sown by scattering on the surface and covering with a thin layer of soil gently compressed over them. A seeding rate of 0.4 g seed/pot was used, equivalent to 630 kg/ha, which ensured full surface coverage and competition between plants in each pot would ensure natural thinning to optimal coverage. Pots were covered with black plastic until germination had started, typically within ~3 days of seed addition. Nutrients were added to the soil according to the rates determined in the preliminary soil analyses described above at two weeks after soil sowing, to allow time for germination and seedling establishment. As plants grew, a clear acetate collar was placed around the pot and moved upwards as the plant grew to ensure vertical growth of herbage and to prevent damage during watering [22].

Five weeks after sowing seeds, plants were harvested to ~2cm above soil level. Stems were rinsed in Milli-Q water to remove any soil contamination. Herbage was frozen, freeze dried, weighed to determine dry matter (DM) yield, and then finely milled. Iodine was analysed using a 25% tetramethylammonium hydroxide (TMAH) extraction for 4 hours with analysis by ICP-MS by NUVetNA (University of Nottingham, UK). Other micronutrient concentrations were determined via a nitric/perchloric digestion of the plant material, followed by ICP-MS and ICP-OES analysis.

### Statistical analysis

Plant yields did not require transformation, but element concentrations and element uptakes (defined as concentration * yield) were transformed with a log_10_ transformation, or a square root transformation in the case of Mo uptake in order to meet the assumptions of the analysis. Statistical analyses were performed in Genstat (VSNi, v20.1.23823) using linear mixed models (LMMs). Each LMM aimed to determine the effect of soil and botanical group, the interaction between soil and botanical group, the effect of plant species within a botanical group, and the interaction between species within a botanical group and soil on yield, and the uptake and concentration of each element individually. To achieve this the fixed model was Soil*(BotanicalGroup/Species). The design structure was accounted for using the random model (Rep/subblock)*Pot. The significance level was set at P < 0.05.

## Results

### Soil characterisation

The chemical properties of soils in which the plants were grown, after nutrient additions, are characterised in Table 1. The pH of the soil ranged over 2 pH units, from 5.4 in the AL to 7.4 in the AH. The NW soil had a SOC of 2.7–3.6% higher than any of the other soils, at 8.7%. The AH and AL soils had extractable P concentrations of 7.5 and 7.8 mg/l respectively, compared with concentrations of 18.7 and 19.9 mg/l in the NW and GM soils respectively. The AL and AH soils had a TON + NH_4_-N content of 147 and 72 kg/ha respectively, compared to 606 and 520 kg/ha in the NW and GM soils.

**Table 1:** Characterisation of the NW (North Wyke, grassland soil), AL (arable low pH), AH (arable high pH) and GM (growing medium) soils after nutrient application.

	Units	NW	AL	AH	GM
pH		5.7 ± 0.09	5.4 ± 0.03	7.4 ± 0.12	6.7 ± 0.09
SOC	% DM	8.7 ± 0.15	6.0 ± 0.10	5.1 ± 0.05	5.6 ± 0.10
Extractable TON + NH_4_-N (KCl extract)	mg/kg soil	155 ± 7	38 ± 1	18 ± 2	133 ± 5
Extractable P (Olsen extract)	mg/l soil	18.7 ± 0.13	7.8 ± 0.72	7.5 ± 0.27	19.9 ± 0.41
Extractable K (ammonium nitrate extract)	mg/l soil	75 ± 0.6	100 ± 2.0	87 ± 1.9	100 ± 1.0
Extractable Mg (ammonium nitrate extract)	mg/l soil	44.7 ± 0.67	53.3 ± 2.19	29.7 ± 0.33	132.0 ± 2.00
Extractable Co (Mehlich extract)	mg/l soil	0.40 ± 0.000	2.13 ± 0.120	2.77 ± 0.067	0.37 ± 0.167
Extractable Cu (Mehlich extract)	mg/l soil	5.77 ± 0.120	3.33 ± 0.067	4.13 ± 0.145	4.13 ± 0.033
Extractable Mn (Mehlich extract)	mg/l soil	80 ± 4.5	254 ± 8.3	307 ± 5.5	32 ± 2.7
Extractable Zn (Mehlich extract)	mg/l soil	6.00 ± 0.100	2.63 ± 0.033	3.87 ± 0.067	3.33 ± 0.570
Extractable Fe (Mehlich extract)	mg/l soil	335 ± 1.3	155 ± 4.3	113 ± 1.7	285 ± 71.9
Extractable SO_4_ (Mehlich extract)	mg/l soil	51 ± 1.7	29 ± 2.7	16 ± 0.8	169 ± 17.9
Total Co	mg/kg soil	7.0 ± 0.11	19.1 ± 0.25	16.3 ± 0.28	7.2 ± 0.21
Total Cu	mg/kg soil	26.4 ± 0.69	14.5 ± 0.16	21.8 ± 3.90	13.5 ± 0.40
Total Mn	mg/kg soil	518 ± 32	1411 ± 13	1309 ± 28	354 ± 10
Total I	mg/kg soil	3.16 ± 0.021	6.43 ± 0.889	5.34 ± 0.041	2.557
Total Se	mg/kg soil	0.86 ± 0.016	0.88 ± 0.017	0.72 ± 0.002	0.48 ± 0.011
Total Zn	mg/kg soil	81 ± 2.2	64 ± 1.1	66 ± 1.1	60 ± 2.7
Total Mo	mg/kg soil	2.54 ± 0.129	0.43 ± 0.014	0.32 ± 0.012	1.09 ± 0.140
Total S	mg/kg soil	334 ± 9.6	127 ± 0.7	131 ± 1.9	221 ± 4.3

The arable soils had higher extractable and total Co, Mn and I concentrations than the other soils, but lower extractable Fe concentrations. Extractable and total Zn concentrations were highest in NW. Extractable and total S was lowest in the arable soils and highest in GM, while total Mo was lowest in the arable soils and highest in NW. Total Se concentrations were lowest in GM.

### Yield

**Fig 1:**
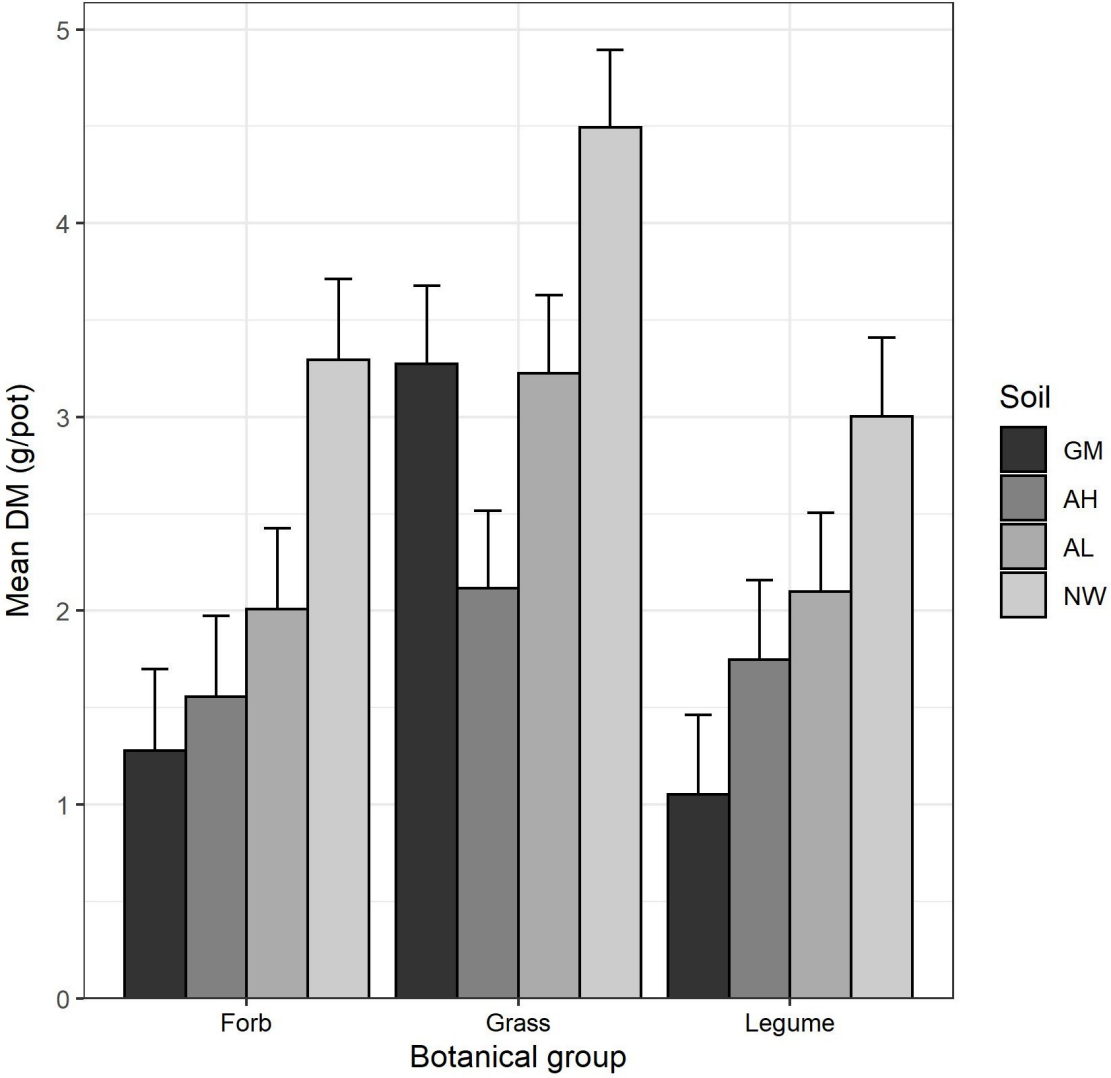
Mean dry matter (DM) yield of botanical groups across the GM, AH, AL and NW soils. Error bars are one standard error. The average least significant difference (LSD) across treatments was 0.497.

Across all soils, there was a significant difference (P < 0.001) in the yield of the botanical groups, with grasses yielding more DM than legumes and forbs (Fig 1). Comparing yield of all species across the different soils (P < 0.001), there was a clear trend of NW > AL > AH > GM. However, there was a significant interaction between soil and botanical group (P < 0.001). All botanical groups had a significantly greater biomass when grown on NW soil than on any other soil. Yields of legumes and forbs then decreased in the order AL > AH > GM, although differences were not always significant. The grasses however grew much better on the GM, with similar yields in the GM and AL soils, both of which were significantly greater than the AH soil.

**Fig 2:**
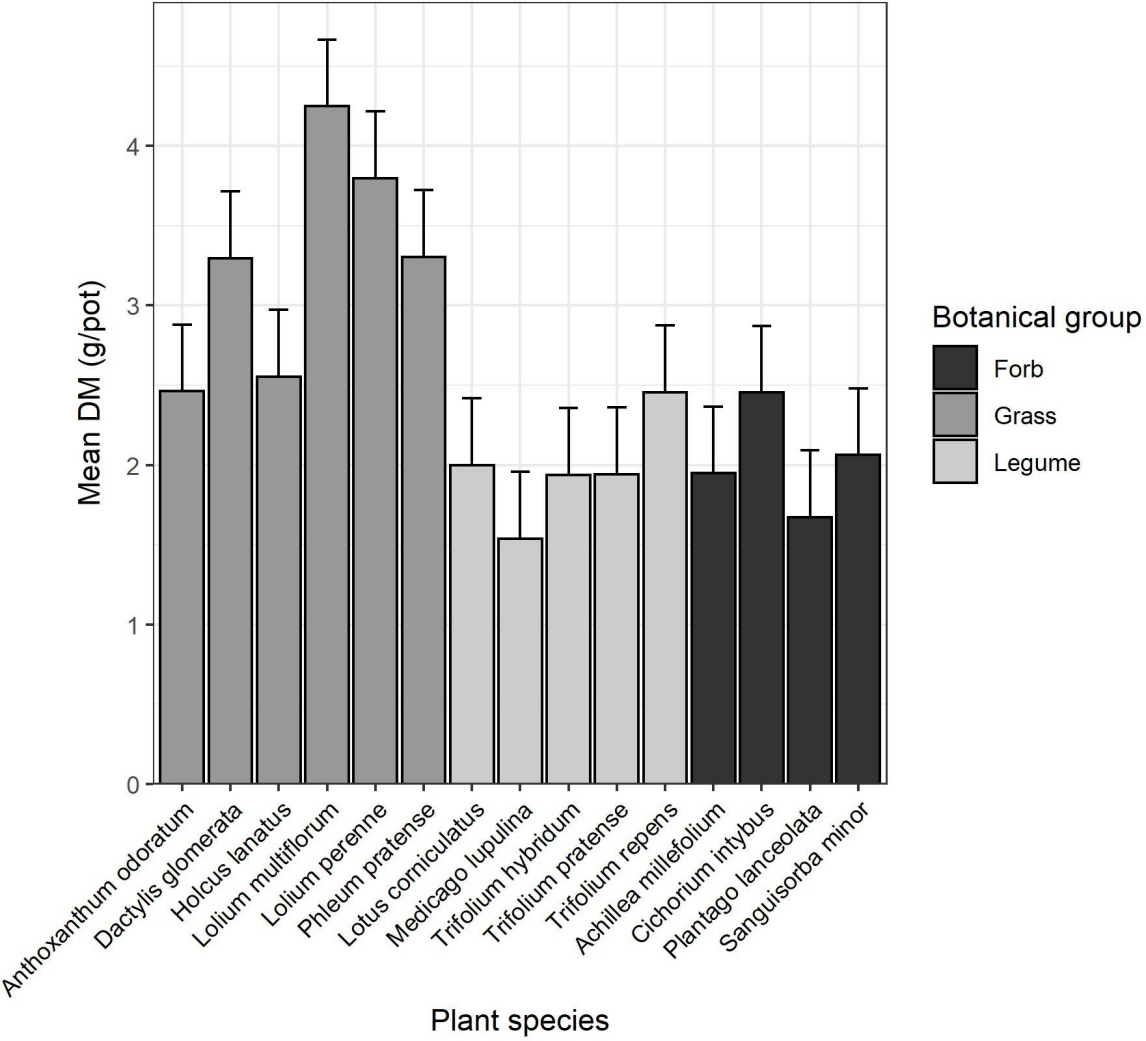
Mean dry matter (DM) yield of species across all of the soil types. Errors bars indicate one standard error. The average least significant difference (LSD) across treatments was 0.491.

Within each botanical group, there was a significant effect of plant species on yield (P = 0.040, <0.001 and 0.029 for forbs, grasses and legumes, respectively; Fig 2). Grasses showed the greatest variability in yield (range 1.54–2.46 g/pot, s.e. 0.418 g/pot), with *L multiflorum* having the greatest yield and *A*. *odoratum* and *H*. *lanatus* the lowest. Legumes varied from 1.54 g/pot, s.e. 0.418 (*M*. *lupulina*) to 2.46 g/pot, s.e. 0.418 (*T*. *repens*), while forbs varied from 1.67 g/pot, s.e. 0.418 (*P*. *lanceolata*) to 2.45 g/pot, s.e. 0.418 (*C*. *intybus*). However, there was no strong interaction between soil and the intra-botanical group variability (P = 0.062, 0.08, 0.966 for forbs, grasses and legumes respectively).

### Effect of soil on plant micronutrient content

The soil on which plants were grown had a significant effect (P ≤ 0.025, see Table 2 for individual results) on both the concentration and the uptake (concentration x DM yield) of each of the micronutrients tested (Co, Cu, Mn, I, Se, Zn, Fe, Mo and S). There was no clear pattern across micronutrients when comparing the plants grown on different soils. However, generally soils with the highest (extractable or total) concentrations of a micronutrient resulted in plants with the highest concentrations of that micronutrient. There were exceptions to this trend, such as the AH soil which had the highest extractable Mn concentration of the soils, but the lowest plant concentrations, while the opposite was the case for total Mo concentrations. Similarly, plant total Se concentrations were highest when grown on the AH and GM soils, but soil Se concentrations were lowest for these soils, being 0.72 and 0.48 mg/kg respectively, compared to 0.86 and 0.88 mg/kg for NW and AL soils respectively.

**Table 2:** Summary of the P values of the REML analysis for the concentration and uptake of each nutrient for soil, botanical group, plant species (within a botanical group) and the interaction of soil with each of these.

Concentration	Co	Cu	Fe	I	Mn	Mo	S	Se	Zn
Soil	0.003	<0.001	<0.001	<0.001	<0.001	<0.001	<0.001	<0.001	<0.001
Botanical group	<0.001	<0.001	<0.001	<0.001	0.596	0.196	<0.001	0.002	<0.001
Soil-botanical group interaction	0.018	0.008	0.126	0.469	0.012	0.281	0.684	0.004	0.021
Forb species	0.001	<0.001	0.003	0.265	0.003	0.125	0.031	0.147	<0.001
Grass species	0.208	0.174	0.392	0.315	<0.001	0.016	0.331	<0.001	0.031
Legume species	0.122	<0.001	0.211	0.499	0.008	0.008	0.368	0.011	<0.001
Soil-forb species interaction	0.633	0.345	0.075	0.479	0.043	0.118	0.236	0.477	0.068
Soil-grass species interaction	0.451	0.509	0.435	0.142	0.231	0.645	0.544	0.842	0.681
Soil-legume species interaction	0.296	0.195	0.908	0.787	0.995	0.905	0.685	0.367	0.55
**Uptake**									
Soil	<0.001	<0.001	<0.001	<0.001	<0.001	<0.001	<0.001	0.025	<0.001
Botanical group	0.018	<0.001	0.004	0.103	<0.001	<0.001	0.054	<0.001	<0.001
Soil-botanical group interaction	0.522	0.006	0.58	0.422	0.538	0.092	0.008	0.005	0.14
Forb species	0.022	<0.001	0.039	0.1	<0.001	0.452	0.064	0.132	<0.001
Grass species	0.043	<0.001	0.167	0.097	0.017	0.093	0.04	0.028	<0.001
Legume species	0.197	0.022	0.053	0.107	0.204	0.004	0.549	0.002	0.037
Soil-forb species interaction	0.265	0.042	0.001	0.119	0.239	0.045	0.027	0.019	0.024
Soil-grass species interaction	0.537	0.328	0.27	0.069	0.182	0.194	0.194	0.668	0.371
Soil-legume species interaction	0.587	0.355	0.172	0.286	0.74	0.803	0.469	0.386	0.276

Plants grown in the NW soil had the highest uptake of most micronutrients. The exceptions were Mo, for which the sequence of uptake was AH > GM ~ NW > AL, and I, where the sequence was GM > other soils.

Comparing the arable soils, plants grown on the AH soil had higher concentrations of Co, Se and Mo, while those grown on the AL soil had higher concentrations of Cu, Mn, Zn and S. When assessing micronutrient uptake, rather than concentration, the same pattern between the arable soils was seen, although the differences were less likely to be significant than they were for micronutrient concentration.

### Botanical group and soil interaction

There was a significant effect of botanical group on the concentrations of Co, Cu, I, Fe, S, Se and Zn in plants (all P < 0.001, except Se, P = 0.002). Legumes and forbs contained the highest concentrations of these micronutrients, and only for Cu and Zn was there a significant difference between these two botanical groups; in both cases the concentrations in legumes were higher than in forbs. Conversely, concentrations of these micronutrients in grasses were significantly lower than at least one of, and sometimes both of, the other botanical groups. The effect of botanical group on plant uptake of micronutrients was significant for Cu, Mn, Mo, Se, Zn (all P < 0.001), Fe (P = 0.004), S (P = 0.008) and Co (P = 0.018). Across all of these micronutrients, the only significant difference between legumes and forbs was in Zn uptake (legumes > forbs). However, the comparison between grasses and the other two botanical groups for micronutrient uptake was much more variable than for micronutrient concentration. Grasses had a significantly lower uptake of Co and Fe than the other botanical groups, but a significantly higher uptake of Cu, Mn, Mo, Se and Zn.

**Fig 3:**
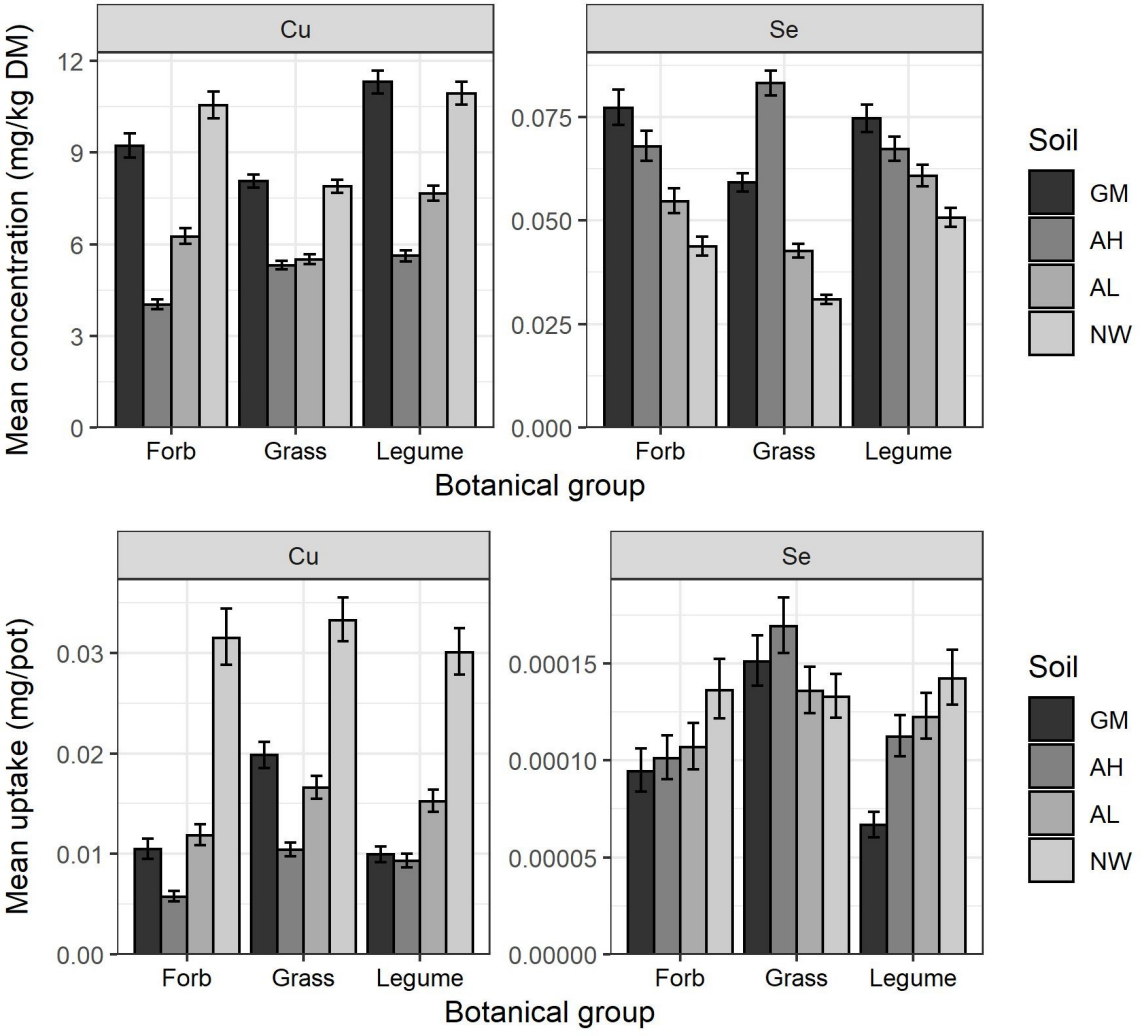
Mean forb, legume and grass Cu concentration and uptake (concentration * plant dry matter yield), and Se concentration and uptake when grown on each of the four soil types: Growing Medium (GM), Arable High pH (AH), Arable Low pH (AL) and North Wyke (NW). Error bars indicate the confidence interval of the back-transformed mean. The least significant difference (LSD) of the back-transformed data were 1.241, 1.421, 1.304 and 1.416 for Cu concentration and uptake and Se concentration and uptake respectively.

Statistical analysis showed a significant interaction between soil and botanical group for Co (P = 0.018), Cu (p = 0.008), Mn (p = 0.012), Se (P = 0.004) and Zn (P = 0.021) concentrations in plants (Se and Cu data are shown in Fig 3, the graphs for the other elements are given in S1–S32 Figs). Comparing the pattern of micronutrient concentrations of the botanical groups across the different soils, the AH soil had higher Co, Cu, Mn and Se concentrations in grasses compared to what may have been predicted in the absence of a soil-botanical group interaction. For example, Se concentrations in the forb and legume groups were highest in the GM soil but were only slightly higher than the AH soil (P > 0.05); in the grasses, the Se concentrations on the AH soil were significantly greater (P < 0.05) than on any of the other soils. Comparing the soil-botanical group results, concentrations of Zn in legumes grown in the AL soil were significantly higher than the AH soil, whereas for the forbs and grasses the difference was not significant. Legume Co concentrations were higher than expected in the GM soil, having a higher concentration than legumes on other soil types (P > 0.05), whereas in the other botanical groups the plants had Co concentrations significantly lower than plants grown on at least one other soil type.

There were fewer significant interactions between soil and botanical group for micronutrient uptake than for micronutrient concentration, although differences were observed for Cu (P = 0.006), Se (P = 0.005) and S (P = 0.008) uptake (Figs 3 and S6). Both Se (P = 0.005) and S (P = 0.008) demonstrate a lower-than-expected uptake by legumes on the GM soil, with, for example, the S uptake by GM soil greater than on the AH and AL soils for both the forbs and grasses, but not significantly different in the legumes.

### Species effect and soil interaction

**Fig 4:**
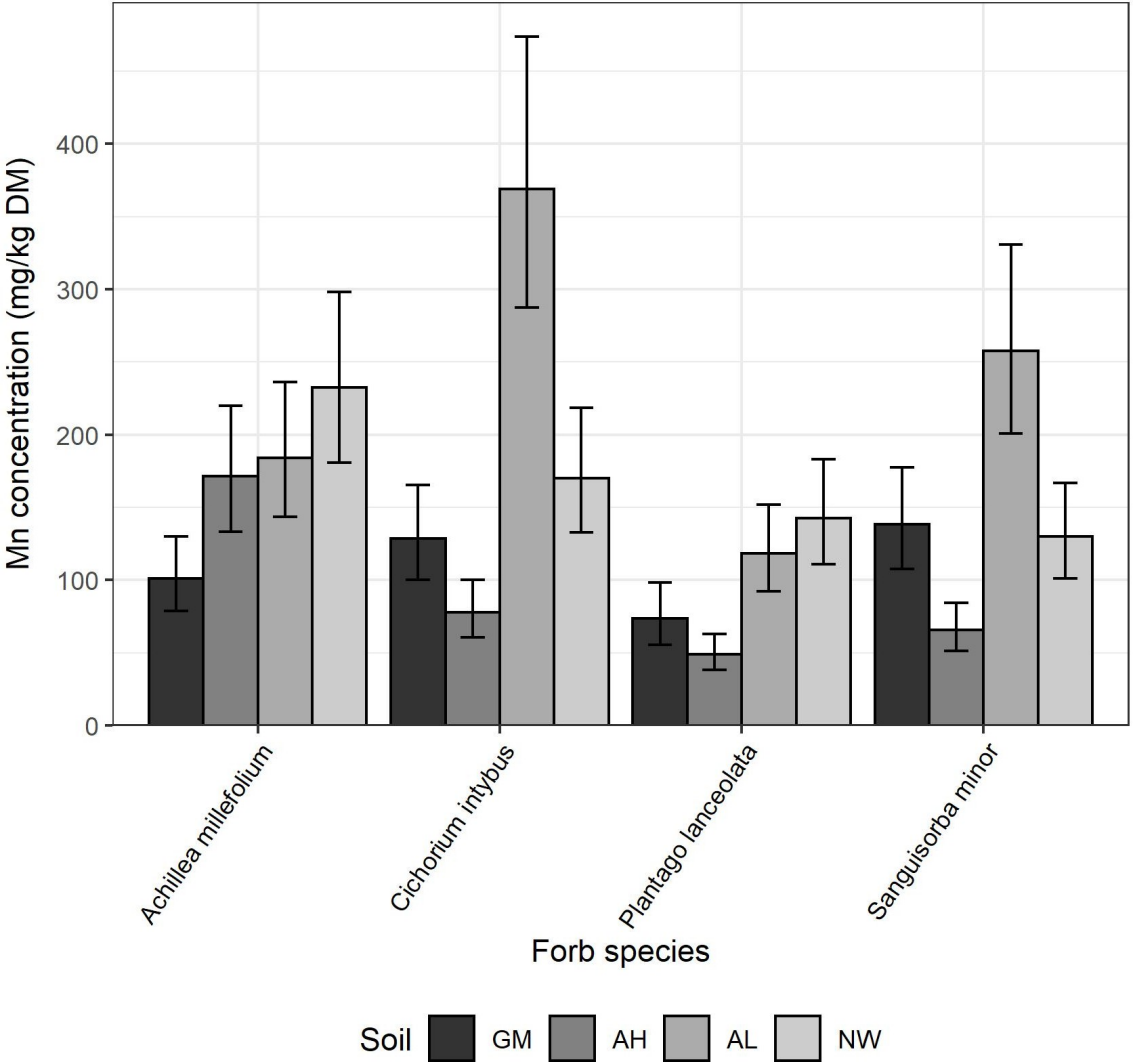
Mean Mn concentration in the plant material of each of the four forb species tested when grown on the Growing Medium (GM), Arable High pH (AH), Arable Low pH (AL) and North Wyke (NW) soils. Error bars are the confidence interval of the back-transformed mean. Average least significant difference (LSD) across all treatments on the back-transformed scale was 2.045.

**Fig 5:**
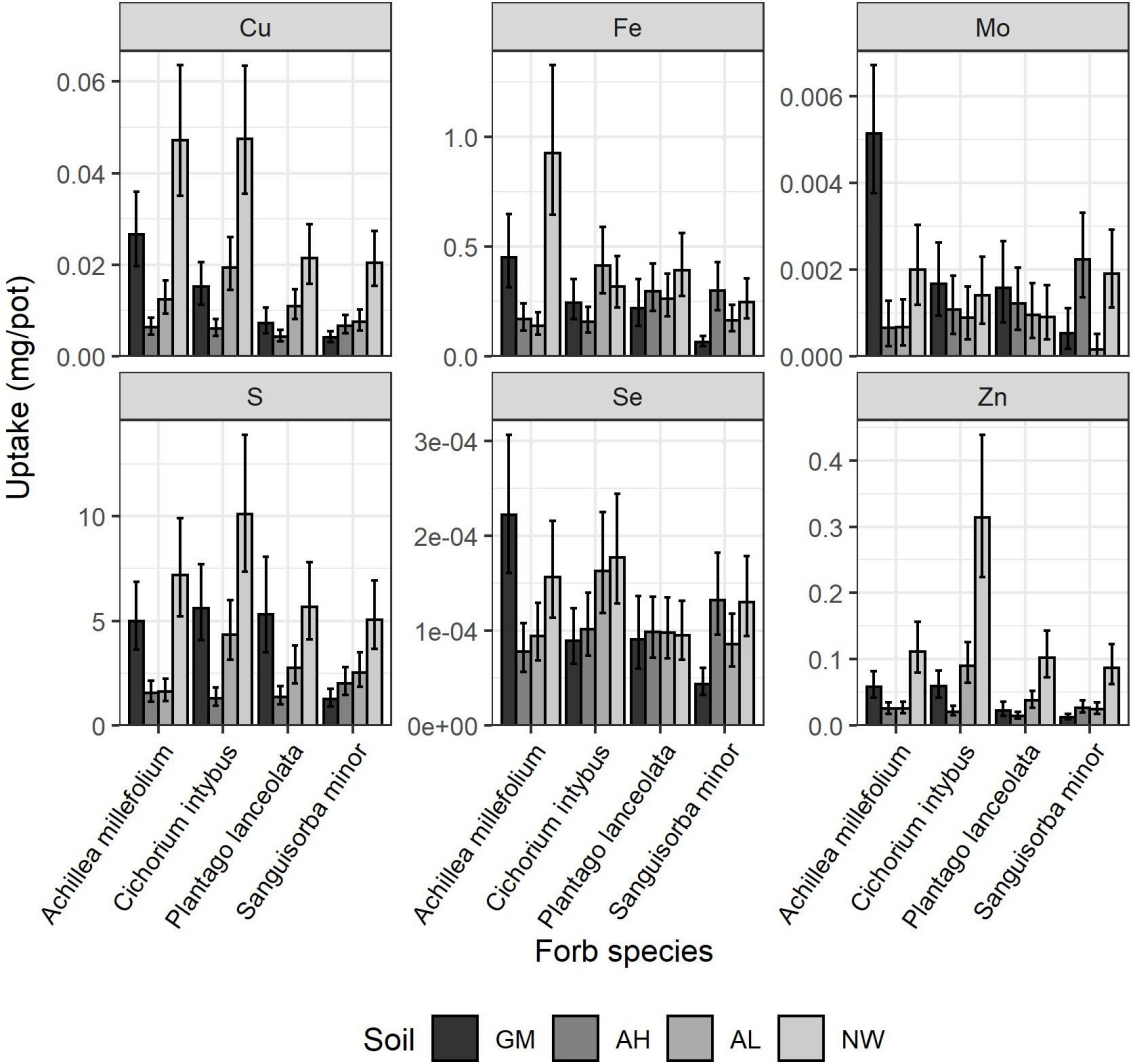
Mean Cu, Fe, Mo, S, Se and Zn uptakes (dry matter yield * concentration) by each of the four forb species tested when grown on the Growing Medium (GM), Arable High pH (AH), Arable Low pH (AL) and North Wyke (NW) soils. Error bars are the confidence interval of the back-transformed mean. Average least significant differences (LSD) of treatments on a back-transformed scale were 2.181, 2.477, 0.0007, 2.314, 2.155 and 2.369 for Cu, Fe, Mo, S, Se and Zn respectively.

Conducting the statistical analysis for each element and botanical group separately, there was often a significant effect of plant species on both the micronutrient concentration in plant material, and in the total uptake of the micronutrient (Table 2). There was no interaction between soil and either grass species or legume species, for either micronutrient concentration or uptake (P > 0.05). However, there was an interaction between soil and forb species for Mn concentration (P = 0.043, Fig 4). In the AH soil, *A*. *millefolium* had a higher Mn concentration than the other forbs, whereas for the GM soil it was *S*. *minor*, in AL it was *C*. *intybus*, and in the NW soil there was no species with a higher Mn concentration, but *P*. *lanceolata* had the lowest concentration. There was a soil-forb species interaction for the uptake of Cu (P = 0.042), Fe (P = 0.001), Mo (P = 0.045), S (P = 0.027), Se (P = 0.019), and Zn (P = 0.024) (Fig 5). Across these micronutrients, it tended to be uptake by *A*. *millefolium* and *C*. *intybus* that led to this interaction. For example, both species had higher Cu uptake than *P*. *lanceolata* and *S*. *minor* in the NW and GM soils, but the differences in Cu uptake between species was less or non-existent in the arable soils. Comparing the arable soils, *A*. *millefolium*, *C*. *intybus* and *P*. *lanceolata* tended to have slightly lower uptake of Mo, Fe and Se when grown in AH than AL, but *S*. *minor* generally had higher uptake of these elements when grown in in AH than AL.

## Discussion

### Effect of soil on plant yield

The soils used in this study were chosen to represent a range of soil conditions in which pasture species may be sown, with the inclusion of the arable soil to acknowledge that pasture leys are often included as part of an arable rotation. The soils showed considerable variation in their pH (5.4–7.4), SOC (5.1–8.7% DM) and their macro- and micro-nutrient concentrations, such that consideration of their effect on yield and micronutrient content of herbage can be extrapolated to other soils, although this could only be tested empirically. Initial soil NPK levels were assessed and amended according to the advice given to UK farmers [18]. That the soil N and P concentrations were still variable after macronutrient addition demonstrates that in different soils these additions are not necessarily translated into equal increases in available nutrients [23], and shows that the soils on which pastures are grown may have very different macronutrient availabilities even when fertilizer recommendations are followed.

The highest DM yield was on the NW soil, explained by good NPK levels, a near-optimal pH for grassland of 5.7 [optimal = pH 6, 24], and a high SOC content, which can improve plant yields [25]. The GM, a growing medium designed for optimal plant growth and with higher N and P levels than the arable soils, might also have been expected to yield well, yet it had the lowest plant yield of all soils. This may reflect differences in the soil structure—the GM treatments required a much greater mass of soil to fill the pots and the dry soil was more akin to a fine powder than a soil with aggregates. Early plant growth on GM was noticeably poorer than on the other soils. In addition to improved root growth, good soil structure leads to better drainage, aeration, and biological turnover of nutrients in the soil [26].

### The best metric for assessing plant micronutrients: Concentration or uptake?

Previous studies into pasture species’ micronutrient concentrations have typically presented plant concentrations as the primary indicator of treatment effects [4], perhaps reporting uptake and/or yield as a secondary measurement [e.g. 27]. This metric is useful because livestock intake requirements for micronutrients are typically reported on a DM concentration basis [28–30], with the assumption that differences in micronutrient requirements across livestock species, breeds and ages are likely to be proportional to DM intake. A previous study has shown that when looking at a single soil type, establishing the relative content of a wide range of pasture species as a micronutrient source led to the same conclusions regardless of whether concentration or uptake was assessed [7]. However, the current study showed considerable differences between the two metrics when growth was over 4 soils. For example, the NW soil generally had the greatest uptake of micronutrients (except Mo and I), whereas herbage concentrations of micronutrients across the soil types showed no clear pattern. Similarly, the soil-forb species interaction was prevalent when looking at micronutrient uptake, but not when considering concentration, and the species with the highest micronutrient contents differed according to which metric it was assessed by.

There were significant differences between the soils with regard to plant yield and also differences in initial micronutrient contents of the soils, and the combination of these factors go some way to explaining why the conclusions drawn often differed between the concentration and uptake metrics. The dilution effect is likely to also play a part, whereby the plant uptake of micronutrients cannot keep pace with the accumulation of DM in fast-growing plants and hence better yielding plants tend to have low concentrations of micronutrients [31]. The dilution effect, noted in numerous studies of both livestock edible and human edible crops [32, 33], means micronutrient concentration and uptake are not independent metrics. By selecting pasture species only on the basis of their micronutrient concentration, it is therefore likely that we may select species that tend to be poor yielding. This is particularly problematic when scaling up the results of a pot study such as this to multispecies swards in the field, where poor yielding species may be only a minor component of the total sward, or potentially may be outcompeted for nutrients, water or light by neighbouring species. Furthermore, our data have demonstrated that different botanical groups grow well in different soils, and therefore the inter-species competition is likely to differ across soils. However, as livestock predominately do not change their intake according to the micronutrient concentrations of the feed, micronutrient concentrations remain an important metric for assessing the quality of pasture for livestock health and productivity. At the pot scale therefore, concentration and uptake data may be equally valuable, whereas at the field scale micronutrient concentrations of the total sward are likely to be the more important metric, as micronutrient uptake by the animal will be determined by DM intake and not, in most systems, by the availability of plant biomass.

### Effect of soil pH on herbage micronutrient concentrations

The herbage grown on the AL soil (pH 5.4) typically had higher concentrations of micronutrients than the herbage grown on the AH soil (pH 7.4), with Co, Se and Mo the exceptions. Differences in soil micronutrient concentrations do not appear to account for this because although concentrations of Cu (Mehlich extractable and total), Mn (Mehlich extractable) and Zn (Mehlich extractable and total) were higher in the AH soil, plant concentrations of these elements were highest in those grown on the AL soil. Similarly, although total Se and Mo concentrations were higher in the AL soil than the AH soil, plants grown on the AH soil had highest concentrations of these elements. However, neither the total nor Mehlich extractable soil concentrations are a true measure of the availability of a nutrient to a plant. Total concentrations include all of the nutrients strongly sorbed to organic or mineral components of the soil that might only be available over long timescales [34], whereas the Mehlich extractant is a better proxy for availability but cannot possibly replicate the intricacies of soil chemistry and plant and microorganism biology that combine to make nutrients available to plants [35]. Furthermore, Mechlich extraction uses an acidic extractant, and therefore may not have had an equal effect on the AH and AL soils. Despite this, the small differences in Mehlich extractable nutrients between the arable soils compared with the GM and NW soils, suggest that it is likely that any differences between the arable soils for soil micronutrient concentrations are within the bounds of the accuracy of the extraction measurement.

Discounting soil micronutrient concentrations as a cause for variability in plant micronutrient concentrations between the arable soils, there are a few other explanations. It is known that as soil pH increases, the availability of most micronutrients (Zn, Cu, Mn, Fe, Co) decreases, while Mo and Se are more available at high pH [36–38]. The dilution effect may be important, as the AL soil consistently yielded better than AH, almost certainly because AL was closer to the optimal pasture soil pH of 6.0 [24]. Furthermore, micro- and macronutrients are not independent of one another in the soil, and there may be positive (synergistic) or negative (antagonistic) effects that affect plant acquisition of micronutrients [39]. Mechanisms include competition for the plasma membrane transporters that take ions with similar chemistry into the roots, for example Zn and Cu, or changes in root exudates due to the presence of one nutrient that can have an effect on another nutrient [39]. Antagonistic and synergistic effects are complex because micronutrients may be affected by the presence of multiple other nutrients, and all effects are mediated by the relative soil concentrations of each nutrient and other soil conditions such as moisture, making predictions challenging [40]. In general, the arable herbage micronutrient concentrations reflected the availability of micronutrients at the soil pH, i.e. Se and Mo highest in AH herbage and Zn, Cu and Mn highest in AL herbage, suggesting this as the dominant mechanism. A similar soil pH response for these three micronutrients in the plant material of *C*. *intybus* has been recorded elsewhere [41]. Contrary to our findings, Crush and Evans [41] found no pH effect on plant yield. Our findings therefore indicate that the dilution effect was not sufficient to outweigh the soil pH effect on micronutrient availability. The exception however is Co, which was generally more available at low soil pH but had the highest concentrations in the AH herbage. From the experimental design it is not possible to determine whether this was due to dilution or to either antagonistic or synergistic effects and remains an area for further study.

Of all the plant species, *S*. *minor* was the only one to yield better on AH than AL. Consequently, it is only for *S*. *minor* that micronutrient uptake of some elements is better on AH than AL. *S*. *minor* is known to prefer neutral to alkaline soils, highlighting the importance of selecting plant species that grow well at the soil pH, for improving overall micronutrient acquisition.

### Should soil affect our choice of species?

As expected, we found that plant yield differed across soils and that the concentrations of micronutrients in herbage often reflected soil concentrations [42]. Furthermore, there are indications that increases in soil organic carbon can decrease the availability of Se to plants, as seen in previous literature [43]. But the key question posed in the introduction was whether soil significantly affects the plants relative to one another, or whether a species with a high micronutrient content in one soil is likely to have a high content in other soils. Previous research into the effect of soil type on the relative concentrations of micronutrients between species within the same botanical group are limited. A comparison of the Co, Cu, Fe, Mn, Mo and Zn concentrations in two grasses (*P*. *pratense*, *Festuca pratensis*) and in two legumes (*T*. *repens*, *T*. *pratense*) found that the relative ranking of the two species within a botanical group differed little across three field sites with contrasting soil properties [9, 37]. When the ranking switched it was where the species had a similar element concentration at one site; the results may also have been confounded by the use of different varieties of a species at different sites [9]. A pot experiment on two contrasting soils with more species (5 grasses, 4 forbs, 3 legumes) found a significant interaction between soil type and plant species for many micronutrients, and was most pronounced for Mn [8]. The relative ranking of the grasses for their Mn concentration did not change across the two soils, but there were changes in relative ranking within the forbs and legumes. As before, the results were affected by the similarity in Mn concentrations between species on one of the soils, where confidence intervals often overlapped slightly or entirely, making it difficult to draw firm conclusions [8]. We also found no evidence to suggest that soil has a significant effect on plant species micronutrient content within either the grasses or the legumes. Interestingly, our data found that the only interaction between soil and forb species for micronutrient concentration was for Mn, echoing the results of Lindstrom et al. [8]. Understanding why this micronutrient is more sensitive to soil variability may be an interesting area for future research.

Unlike previous studies, we also looked at soil effect on micronutrient uptake and found it to have a significant interaction with forb species for many micronutrients. There were fewer forb species tested than legume or grass species, meaning that statistical differences between the species would have been harder to detect in this botanical group. This suggests that it is the selection of the forb species for a multispecies mix that needs to be most soil-specific, especially as it is this botanical group that is most likely to be incorporated into swards for their micronutrient content [44]. Grasses are not a major source of micronutrients [7] and are included in swards for their digestible energy and protein, and although legumes are also good sources of micronutrients, they are predominantly utilised for their N fixing abilities and as a protein source [44]. The effect of soil on the relative micronutrient uptake of species, rather than their concentrations, indicates that attention should be paid to the expected yield of the forbs in the soil. This is particularly important as forbs tend to be a relatively minor component of the total sward compared to grasses and legumes [e.g. 45], and therefore any change in yield will have a disproportionately large effect on uptake of micronutrients by forbs. The significant interaction between soil and botanical group on the yield, concentration and uptake of many micronutrients indicates that this is highly probable and highlights that competition between plants may need to be considered on different soils.

It is not the aim of this paper to make in-depth recommendations of plant species for inclusion in multi-species mixtures. Ideally seed mixtures will be site specific, focusing on the micronutrient(s) most deficient in swards and focused on the requirements of the livestock that the herbage will feed. Depending on the micronutrient(s) of focus, different species may be optimal. Our previous results suggest that good ‘all-rounder’ species for micronutrient delivery are the forbs *A*. *millfolium*, *C*. *intybus* and *P*. *lanceolata*, and the legumes *M*. *lupulina*, *T*. *hybridum* and *L*. *corniculatus* [7], and the current results broadly agree.

### Scaling up the results

A pot scale experiment with controlled environmental variables means that the effect of soil on micronutrient acquisition by plants was free of many confounding variables. But the nature of it means that there are some factors that are therefore not similar to real-world conditions. Most obviously, to prevent roots becoming pot-bound, plants were only grown for five weeks. It is known that herbage micronutrient concentrations tend to decline as a plant matures [31], and therefore the sward that livestock would graze is likely to have lower micronutrient concentrations than we have measured in this experiment. What is less certain is whether the relative ranking of species would be affected by any temporal changes in micronutrient concentrations with plant maturity. Our experimental results are from monocultures, but species in multispecies swards will have to compete with one another for light, water and nutrients which can affect yield, nutrient uptake, and the balance of species in a sward [46]. In the real-world, environmental effects cannot easily be separated from soil effects. Crush et al. [10] found that there was a considerable reranking of *L*. *perenne* cultivars across 4 sites as a consequence of location, season and N fertilizer treatment, although these effects may be reduced in more disparate pasture species than in different varieties of a single species. As previously discussed, the soil-botanical group interaction means that different botanical groups will have a slight advantage on different soils and this can drive overall sward concentrations of micronutrients. For example, Lindstrom et al. [9] found that across three sites planted with the same seed mixes there was a positive correlation between micronutrient content and the proportion of *T*. *pratense* in the mixture. The effect of slurry application to a multi-species sward is to increase the proportion of grasses and consequently reduce the concentration of some nutrients in the sward [47]. Furthermore, a pot study does not take account of temporal differences in germination and growth of species, such as clover typically growing later in the season than grasses, and that micronutrient content of pasture species tends to decline with phenological development [27]. Therefore, further research is required to validate our findings at the field scale and understand how important soil effects are in relation to environmental and land management effects.

## Conclusions

Our results suggest that when considering legume and grass species to include in multispecies swards, it is unnecessary to consider the effect of soil properties on micronutrient acquisition, particularly for grasses which tended to have low concentrations relative to the other botanical groups. However, more consideration needs to be given to forb species, with our data indicating that selection of species that yield well on a specific soil is the key consideration for improving micronutrient uptake. Furthermore, forbs are typically a minor component of multispecies swards and a plant that yields well is also likely to be more competitive in a mixed sward. Further work needs to be done to determine how soil affects micronutrient content of plants in a mixed sward, as our data showed that soil affected both the yield and the micronutrient concentration of the different botanical groups relative to one another.

Soil pH affected both the yield and micronutrient content of plants, both of which were typically higher at pH 5.4 than 7.4. There was an indication that soil pH can result in differences in the relative micronutrient uptake of forb species but were largely due to *S*. *minor* yielding well on the more alkaline soil. This again highlights the importance of considering expected yields when selecting forb species for micronutrient content.

## Supporting information

S1 Fig Mean forb, legume and grass Co concentration when grown on each of the four soil types: Growing Medium (GM), Arable High pH (AH), Arable Low pH (AL) and North Wyke (NW). Error bars indicate the confidence interval of the back-transformed mean.
(JPEG)

S2 Fig Mean forb, legume and grass Fe concentration when grown on each of the four soil types: Growing Medium (GM), Arable High pH (AH), Arable Low pH (AL) and North Wyke (NW). Error bars indicate the confidence interval of the back-transformed mean.
(JPEG)

S3 Fig Mean forb, legume and grass I concentration when grown on each of the four soil types: Growing Medium (GM), Arable High pH (AH), Arable Low pH (AL) and North Wyke (NW). Error bars indicate the confidence interval of the back-transformed mean.
(JPEG)

S4 Fig Mean forb, legume and grass Mn concentration when grown on each of the four soil types: Growing Medium (GM), Arable High pH (AH), Arable Low pH (AL) and North Wyke (NW). Error bars indicate the confidence interval of the back-transformed mean.
(JPEG)

S5 Fig Mean forb, legume and grass Mo concentration when grown on each of the four soil types: Growing Medium (GM), Arable High pH (AH), Arable Low pH (AL) and North Wyke (NW). Error bars indicate the confidence interval of the back-transformed mean.
(JPEG)

S6 Fig Mean forb, legume and grass S concentration when grown on each of the four soil types: Growing Medium (GM), Arable High pH (AH), Arable Low pH (AL) and North Wyke (NW). Error bars indicate the confidence interval of the back-transformed mean.
(JPEG)

S7 Fig Mean forb, legume and grass Zn concentration when grown on each of the four soil types: Growing Medium (GM), Arable High pH (AH), Arable Low pH (AL) and North Wyke (NW). Error bars indicate the confidence interval of the back-transformed mean.
(JPEG)

S8 Fig Mean forb, legume and grass Co uptake when grown on each of the four soil types: Growing Medium (GM), Arable High pH (AH), Arable Low pH (AL) and North Wyke (NW). Error bars indicate the confidence interval of the back-transformed mean.
(JPEG)

S9 Fig Mean forb, legume and grass Fe uptake when grown on each of the four soil types: Growing Medium (GM), Arable High pH (AH), Arable Low pH (AL) and North Wyke (NW). Error bars indicate the confidence interval of the back-transformed mean.
(JPEG)

S10 Fig Mean forb, legume and grass I uptake when grown on each of the four soil types: Growing Medium (GM), Arable High pH (AH), Arable Low pH (AL) and North Wyke (NW). Error bars indicate the confidence interval of the back-transformed mean.
(JPEG)

S11 Fig Mean forb, legume and grass Mn uptake when grown on each of the four soil types: Growing Medium (GM), Arable High pH (AH), Arable Low pH (AL) and North Wyke (NW). Error bars indicate the confidence interval of the back-transformed mean.
(JPEG)

S12 Fig Mean forb, legume and grass Mo uptake when grown on each of the four soil types: Growing Medium (GM), Arable High pH (AH), Arable Low pH (AL) and North Wyke (NW). Error bars indicate the confidence interval of the back-transformed mean.
(JPEG)

S13 Fig Mean forb, legume and grass S uptake when grown on each of the four soil types: Growing Medium (GM), Arable High pH (AH), Arable Low pH (AL) and North Wyke (NW). Error bars indicate the confidence interval of the back-transformed mean.
(JPEG)

S14 Fig Mean forb, legume and grass Zn uptake when grown on each of the four soil types: Growing Medium (GM), Arable High pH (AH), Arable Low pH (AL) and North Wyke (NW). Error bars indicate the confidence interval of the back-transformed mean.
(JPEG)

S15 Fig Mean Co concentration in the plant material of each species when grown on each of the four soil types: Growing Medium (GM), Arable High pH (AH), Arable Low pH (AL) and North Wyke (NW). Error bars indicate the confidence interval of the back-transformed mean.
(JPEG)

S16 Fig Mean Cu concentration in the plant material of each species when grown on each of the four soil types: Growing Medium (GM), Arable High pH (AH), Arable Low pH (AL) and North Wyke (NW). Error bars indicate the confidence interval of the back-transformed mean.
(JPEG)

S17 Fig Mean Fe concentration in the plant material of each species when grown on each of the four soil types: Growing Medium (GM), Arable High pH (AH), Arable Low pH (AL) and North Wyke (NW). Error bars indicate the confidence interval of the back-transformed mean.
(JPEG)

S18 Fig Mean I concentration in the plant material of each species when grown on each of the four soil types: Growing Medium (GM), Arable High pH (AH), Arable Low pH (AL) and North Wyke (NW). Error bars indicate the confidence interval of the back-transformed mean.
(JPEG)

S19 Fig Mean Mn concentration in the plant material of each species when grown on each of the four soil types: Growing Medium (GM), Arable High pH (AH), Arable Low pH (AL) and North Wyke (NW). Error bars indicate the confidence interval of the back-transformed mean.
(JPEG)

S20 Fig Mean Mo concentration in the plant material of each species when grown on each of the four soil types: Growing Medium (GM), Arable High pH (AH), Arable Low pH (AL) and North Wyke (NW). Error bars indicate the confidence interval of the back-transformed mean.
(JPEG)

S21 Fig Mean S concentration in the plant material of each species when grown on each of the four soil types: Growing Medium (GM), Arable High pH (AH), Arable Low pH (AL) and North Wyke (NW). Error bars indicate the confidence interval of the back-transformed mean.
(JPEG)

S22 Fig Mean Se concentration in the plant material of each species when grown on each of the four soil types: Growing Medium (GM), Arable High pH (AH), Arable Low pH (AL) and North Wyke (NW). Error bars indicate the confidence interval of the back-transformed mean.
(JPEG)

S23 Fig Mean Zn concentration in the plant material of each species when grown on each of the four soil types: Growing Medium (GM), Arable High pH (AH), Arable Low pH (AL) and North Wyke (NW). Error bars indicate the confidence interval of the back-transformed mean.
(JPEG)

S24 Fig Mean Co uptake by each species when grown on each of the four soil types: Growing Medium (GM), Arable High pH (AH), Arable Low pH (AL) and North Wyke (NW). Error bars indicate the confidence interval of the back-transformed mean.
(JPEG)

S25 Fig Mean Cu uptake by each species when grown on each of the four soil types: Growing Medium (GM), Arable High pH (AH), Arable Low pH (AL) and North Wyke (NW). Error bars indicate the confidence interval of the back-transformed mean.
(JPEG)

S26 Fig Mean Fe uptake by each species when grown on each of the four soil types: Growing Medium (GM), Arable High pH (AH), Arable Low pH (AL) and North Wyke (NW). Error bars indicate the confidence interval of the back-transformed mean.
(JPEG)

S27 Fig Mean I uptake by each species when grown on each of the four soil types: Growing Medium (GM), Arable High pH (AH), Arable Low pH (AL) and North Wyke (NW). Error bars indicate the confidence interval of the back-transformed mean.
(JPEG)

S28 Fig Mean Mn uptake by each species when grown on each of the four soil types: Growing Medium (GM), Arable High pH (AH), Arable Low pH (AL) and North Wyke (NW). Error bars indicate the confidence interval of the back-transformed mean.
(JPEG)

S29 Fig Mean Mo uptake by each species when grown on each of the four soil types: Growing Medium (GM), Arable High pH (AH), Arable Low pH (AL) and North Wyke (NW). Error bars indicate the confidence interval of the back-transformed mean.
(JPEG)

S30 Fig Mean S uptake by each species when grown on each of the four soil types: Growing Medium (GM), Arable High pH (AH), Arable Low pH (AL) and North Wyke (NW). Error bars indicate the confidence interval of the back-transformed mean.
(JPEG)

S31 Fig Mean Se uptake by each species when grown on each of the four soil types: Growing Medium (GM), Arable High pH (AH), Arable Low pH (AL) and North Wyke (NW). Error bars indicate the confidence interval of the back-transformed mean.
(JPEG)

S32 Fig Mean Zn uptake by each species when grown on each of the four soil types: Growing Medium (GM), Arable High pH (AH), Arable Low pH (AL) and North Wyke (NW). Error bars indicate the confidence interval of the back-transformed mean.
(JPEG)
