# Supplementary figures and images for: The effect of soil type on yield and micronutrient content of pasture species
Source: PLoS One. 2022 Nov 2;17(11):e0277091. doi: 10.1371/journal.pone.0277091 (PMC9629613; doi:10.1371/journal.pone.0277091)

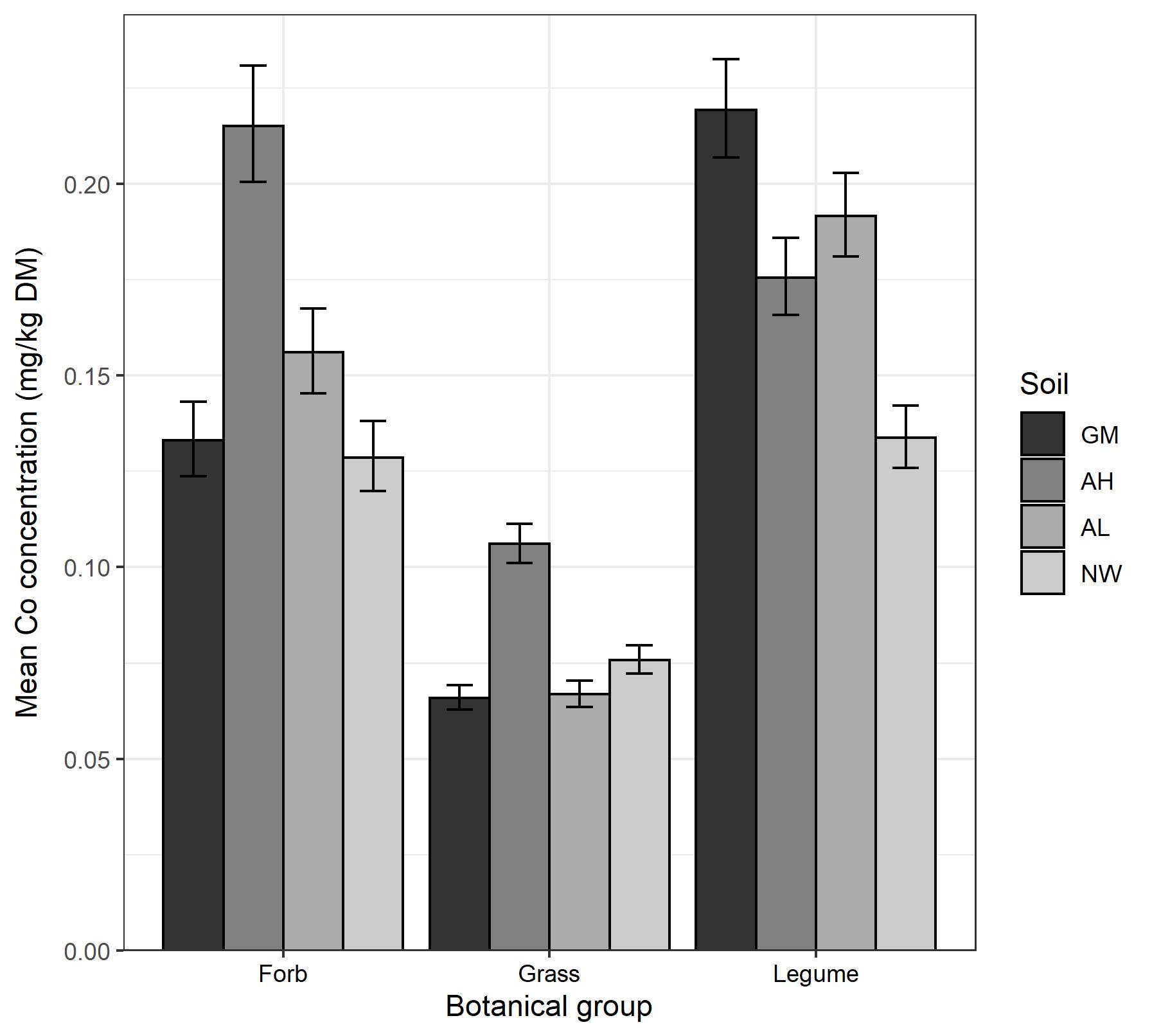

Supplement: S1 Fig — Error bars indicate the confidence interval of the back-transformed mean. (JPEG) [file pone.0277091.s001.jpeg]

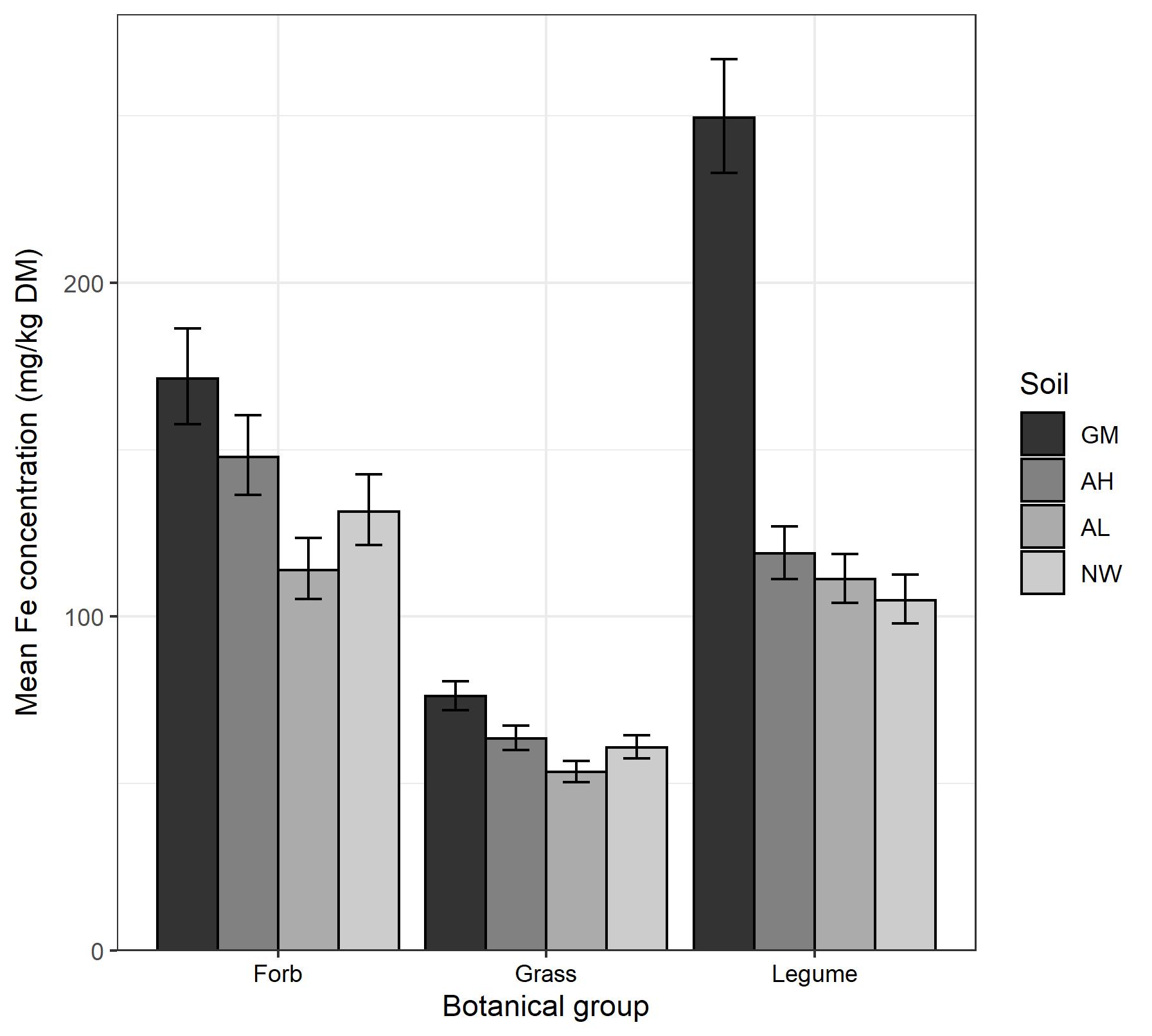

Supplement: S2 Fig — Error bars indicate the confidence interval of the back-transformed mean. (JPEG) [file pone.0277091.s002.jpeg]

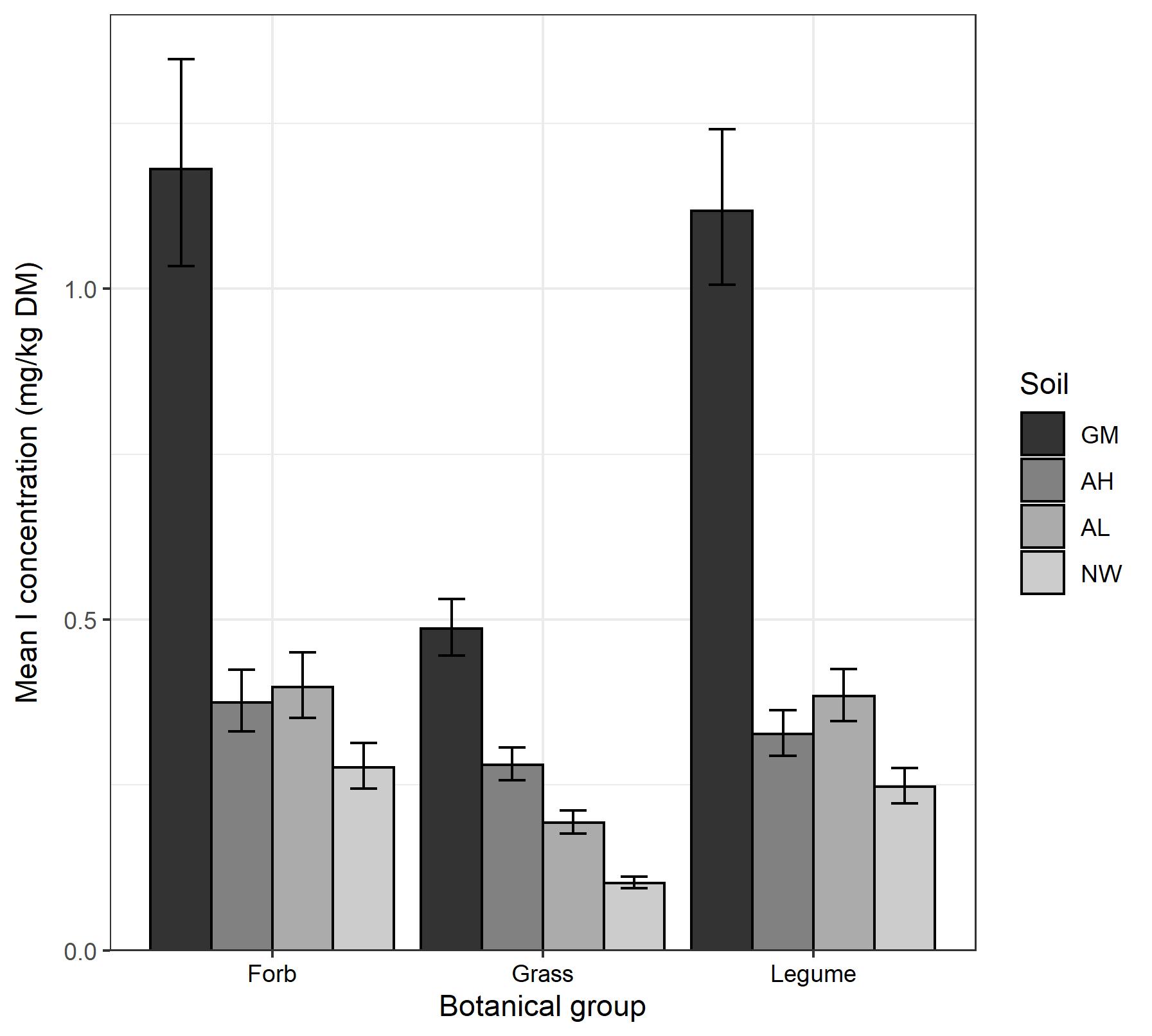

Supplement: S3 Fig — Error bars indicate the confidence interval of the back-transformed mean. (JPEG) [file pone.0277091.s003.jpeg]

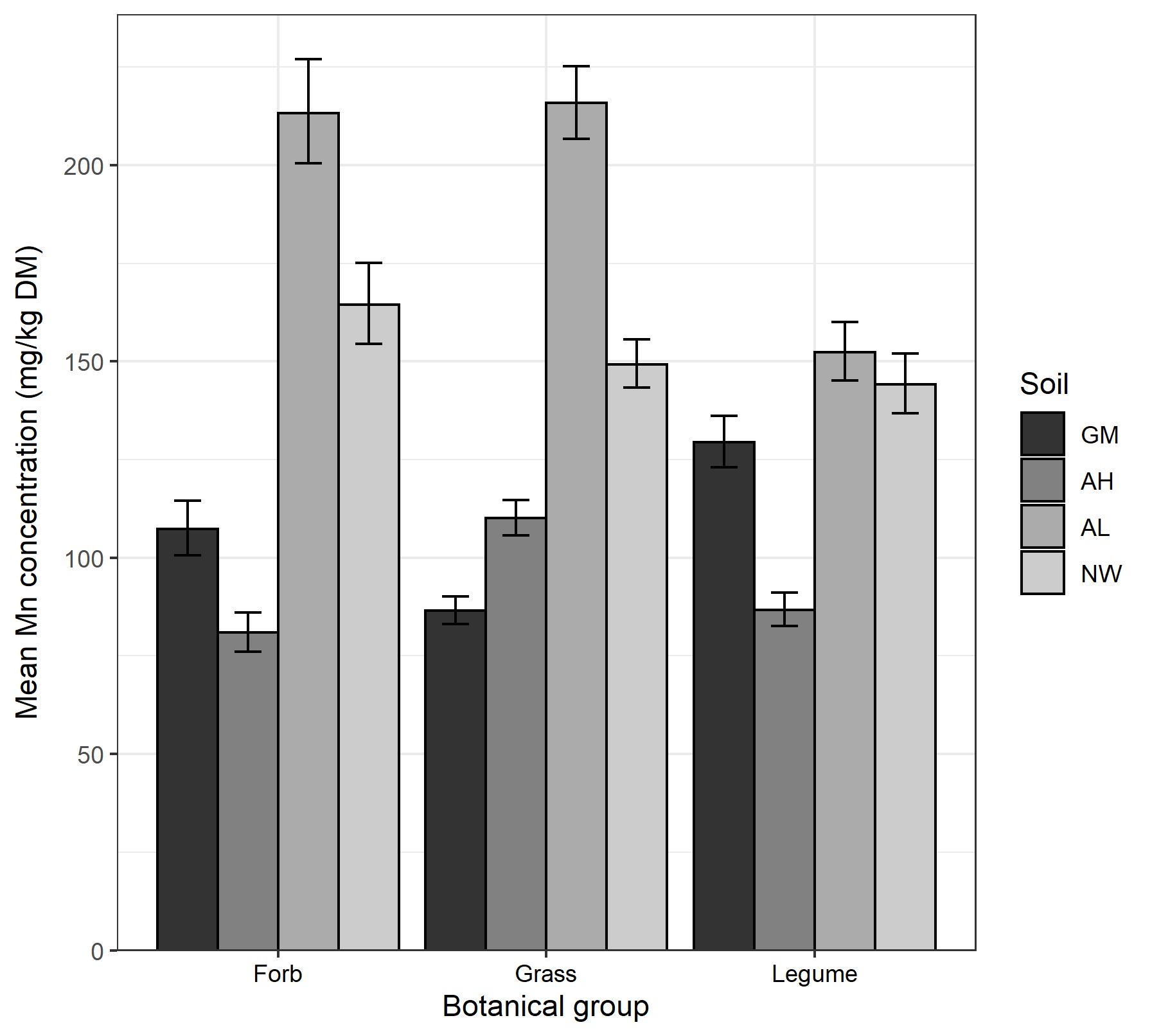

Supplement: S4 Fig — Error bars indicate the confidence interval of the back-transformed mean. (JPEG) [file pone.0277091.s004.jpeg]

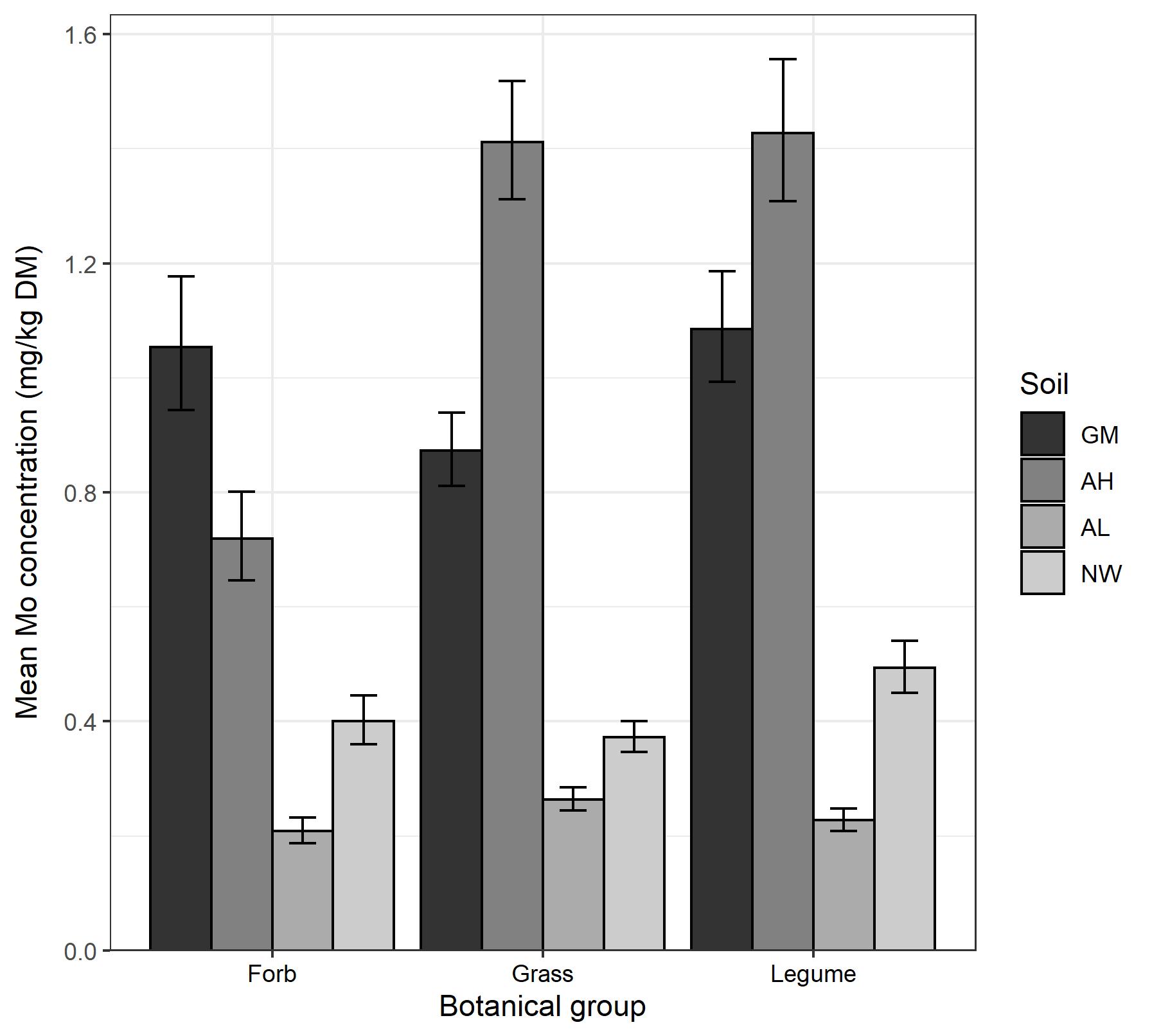

Supplement: S5 Fig — Error bars indicate the confidence interval of the back-transformed mean. (JPEG) [file pone.0277091.s005.jpeg]

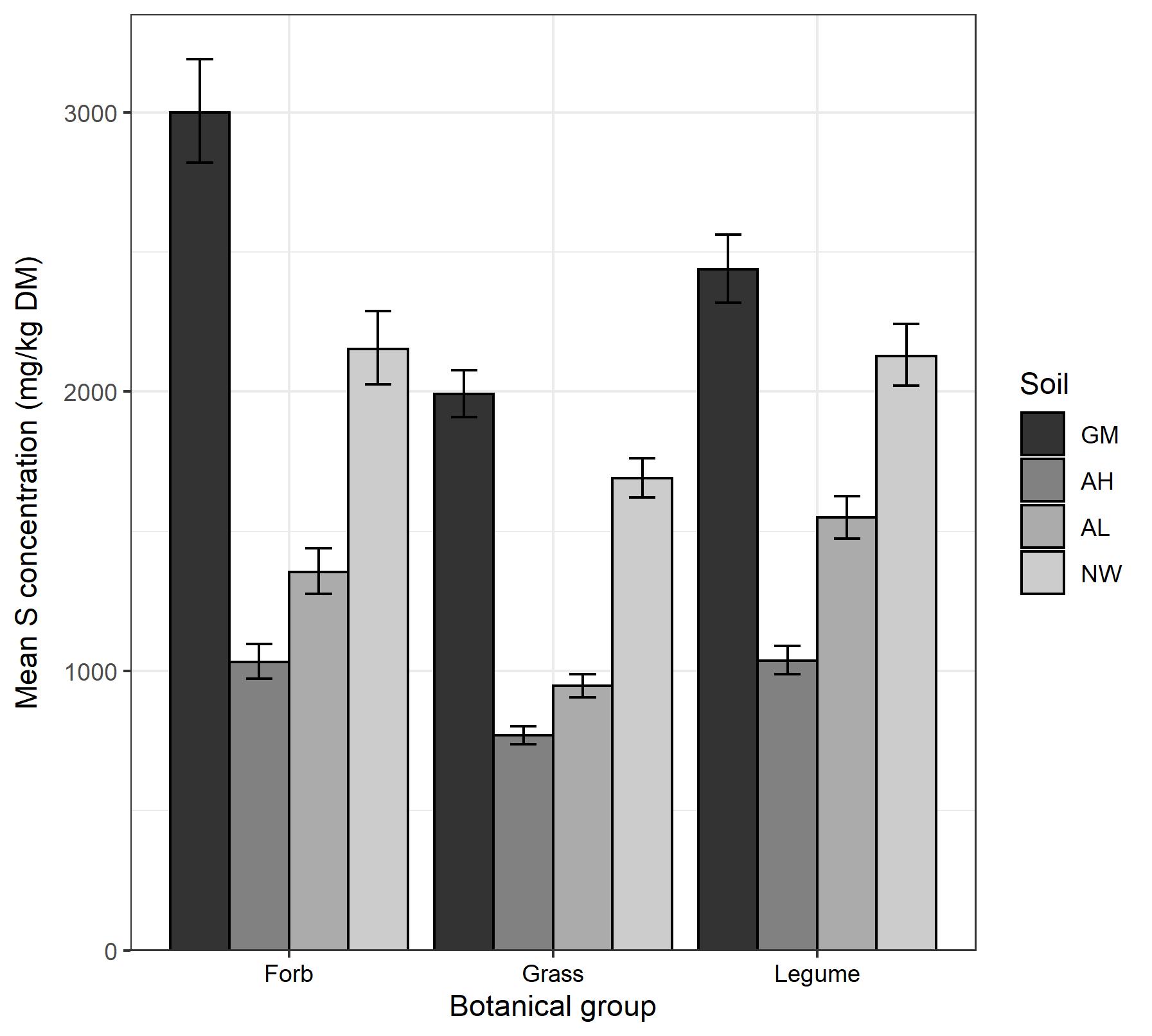

Supplement: S6 Fig — Error bars indicate the confidence interval of the back-transformed mean. (JPEG) [file pone.0277091.s006.jpeg]

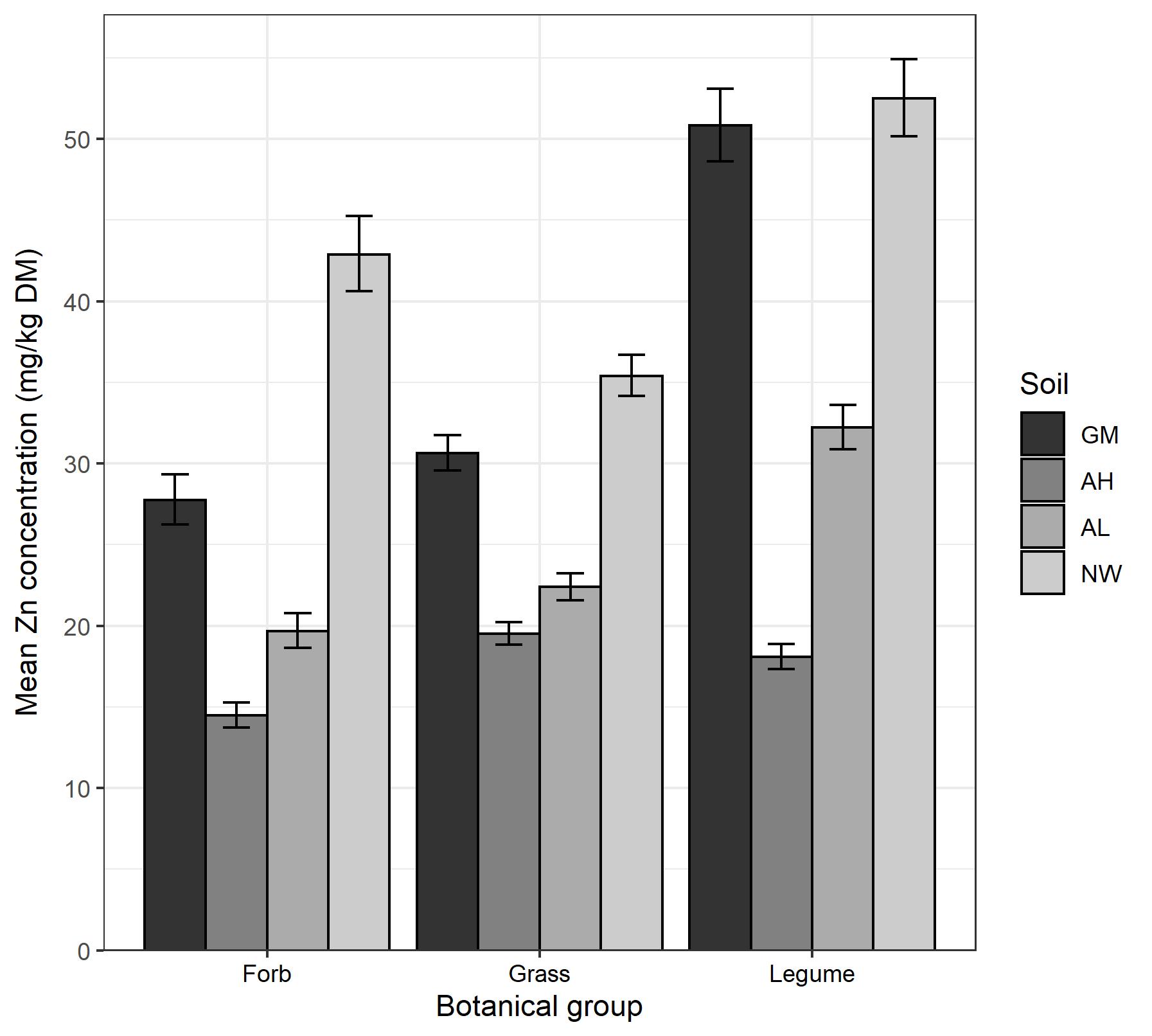

Supplement: S7 Fig — Error bars indicate the confidence interval of the back-transformed mean. (JPEG) [file pone.0277091.s007.jpeg]

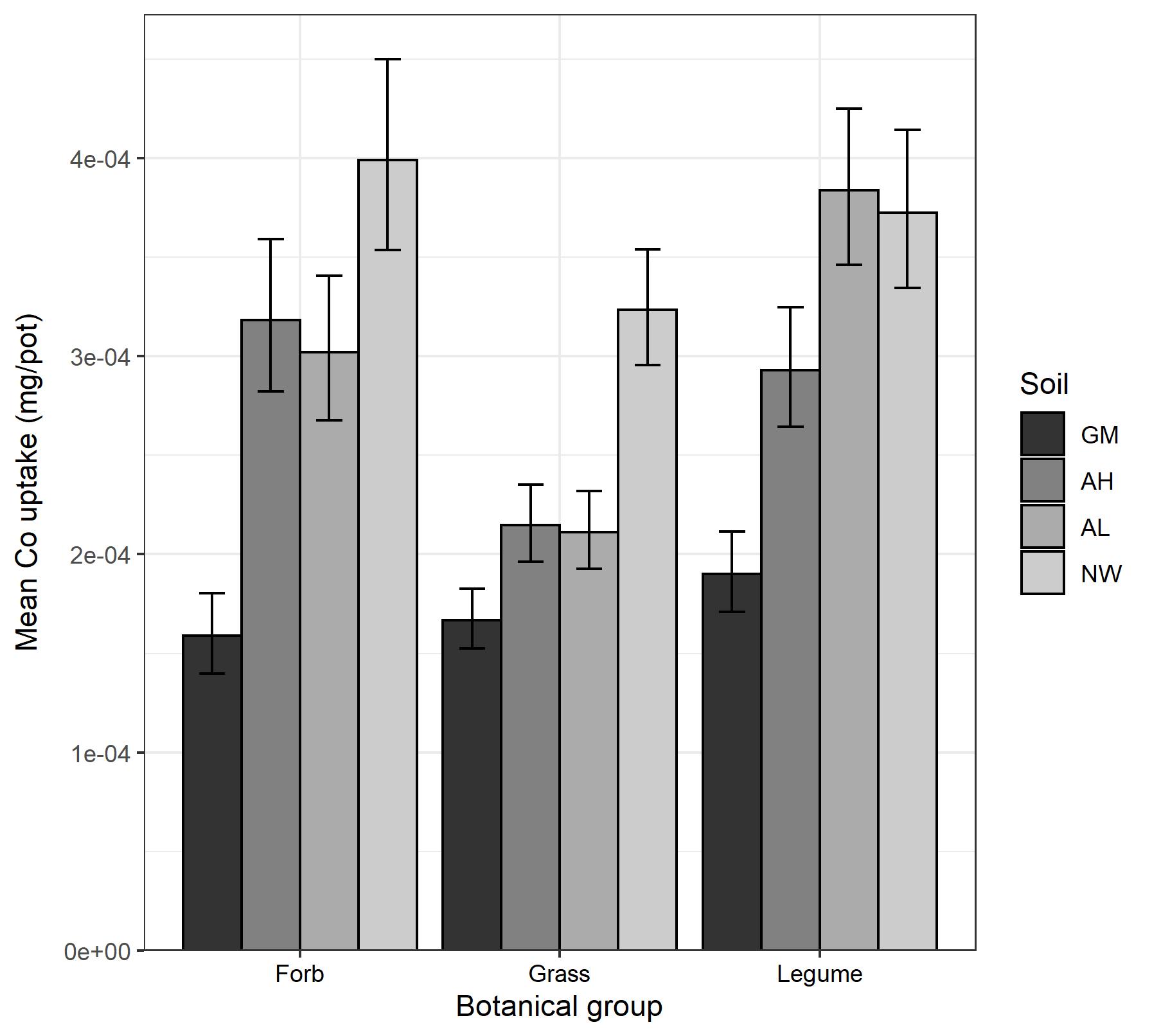

Supplement: S8 Fig — Error bars indicate the confidence interval of the back-transformed mean. (JPEG) [file pone.0277091.s008.jpeg]

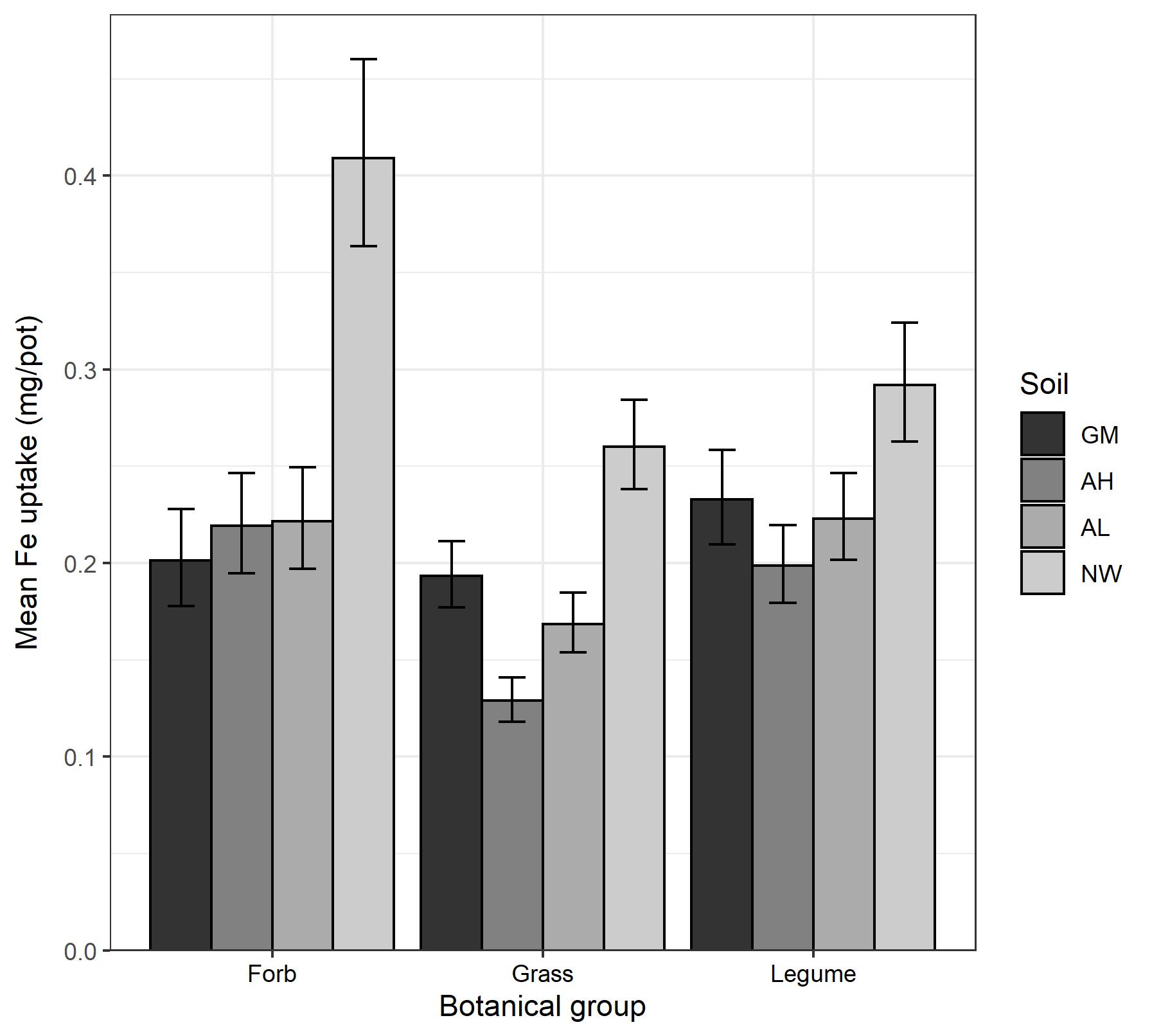

Supplement: S9 Fig — Error bars indicate the confidence interval of the back-transformed mean. (JPEG) [file pone.0277091.s009.jpeg]

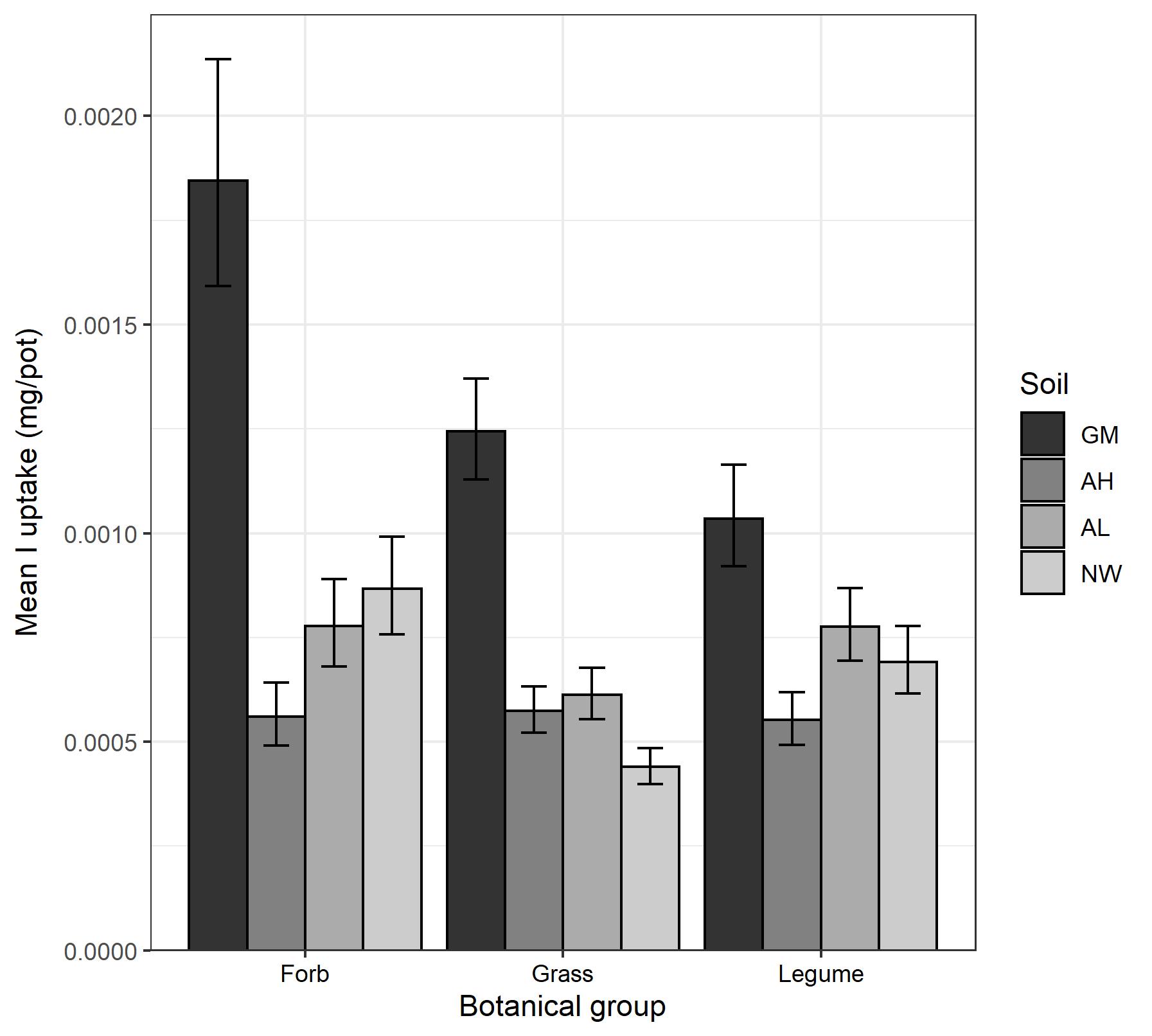

Supplement: S10 Fig — Error bars indicate the confidence interval of the back-transformed mean. (JPEG) [file pone.0277091.s010.jpeg]

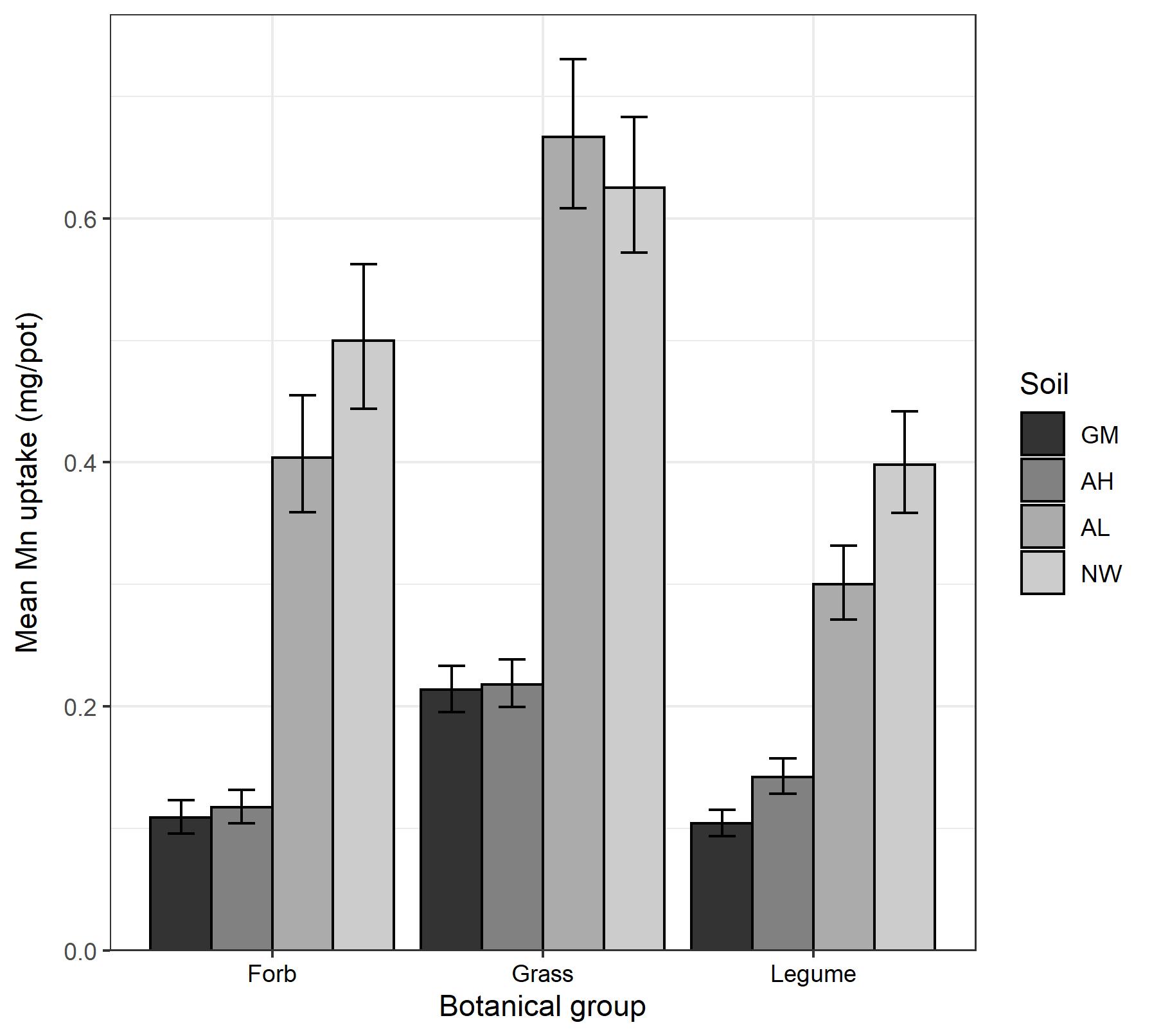

Supplement: S11 Fig — Error bars indicate the confidence interval of the back-transformed mean. (JPEG) [file pone.0277091.s011.jpeg]

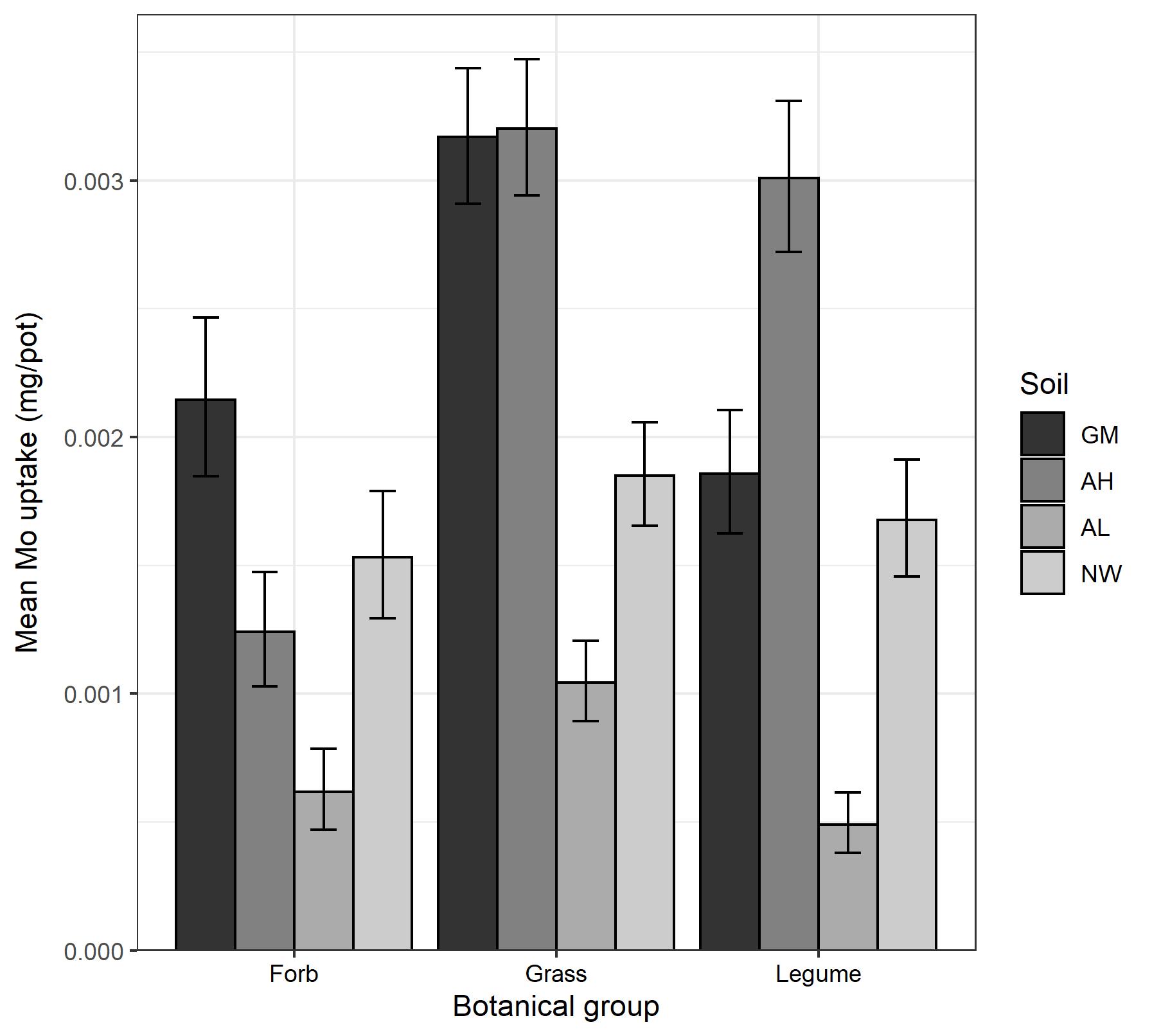

Supplement: S12 Fig — Error bars indicate the confidence interval of the back-transformed mean. (JPEG) [file pone.0277091.s012.jpeg]

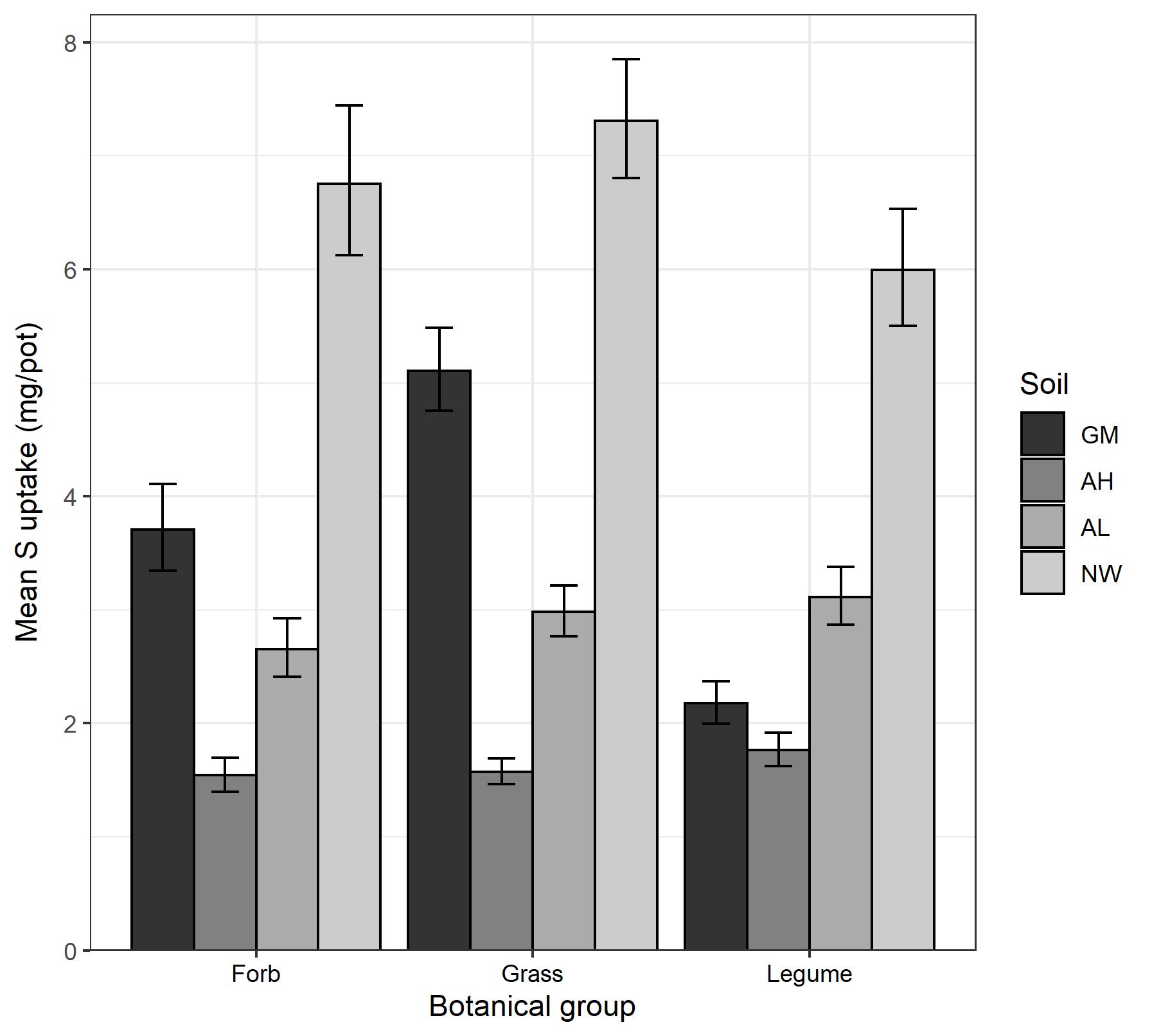

Supplement: S13 Fig — Error bars indicate the confidence interval of the back-transformed mean. (JPEG) [file pone.0277091.s013.jpeg]

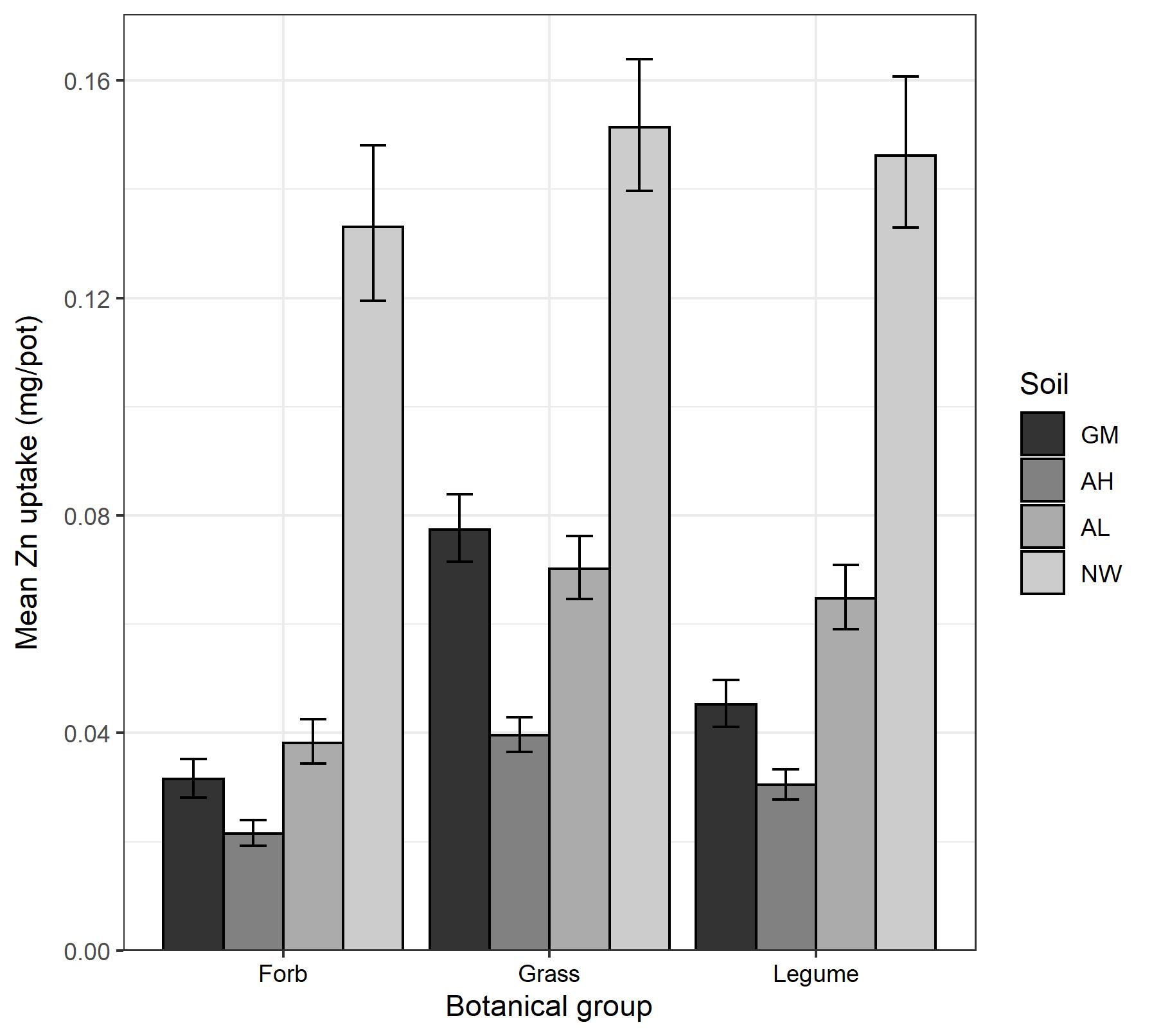

Supplement: S14 Fig — Error bars indicate the confidence interval of the back-transformed mean. (JPEG) [file pone.0277091.s014.jpeg]

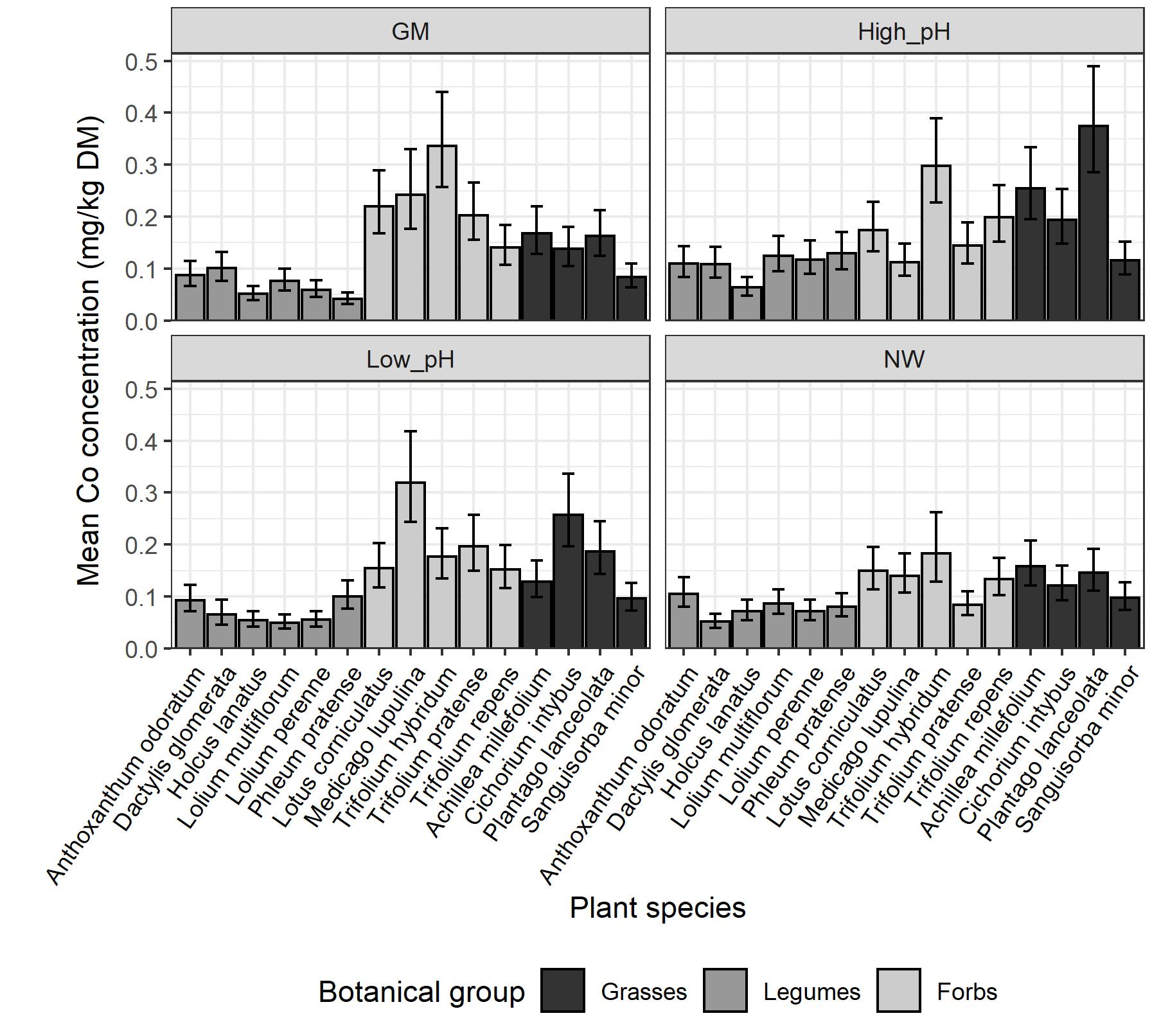

Supplement: S15 Fig — Error bars indicate the confidence interval of the back-transformed mean. (JPEG) [file pone.0277091.s015.jpeg]

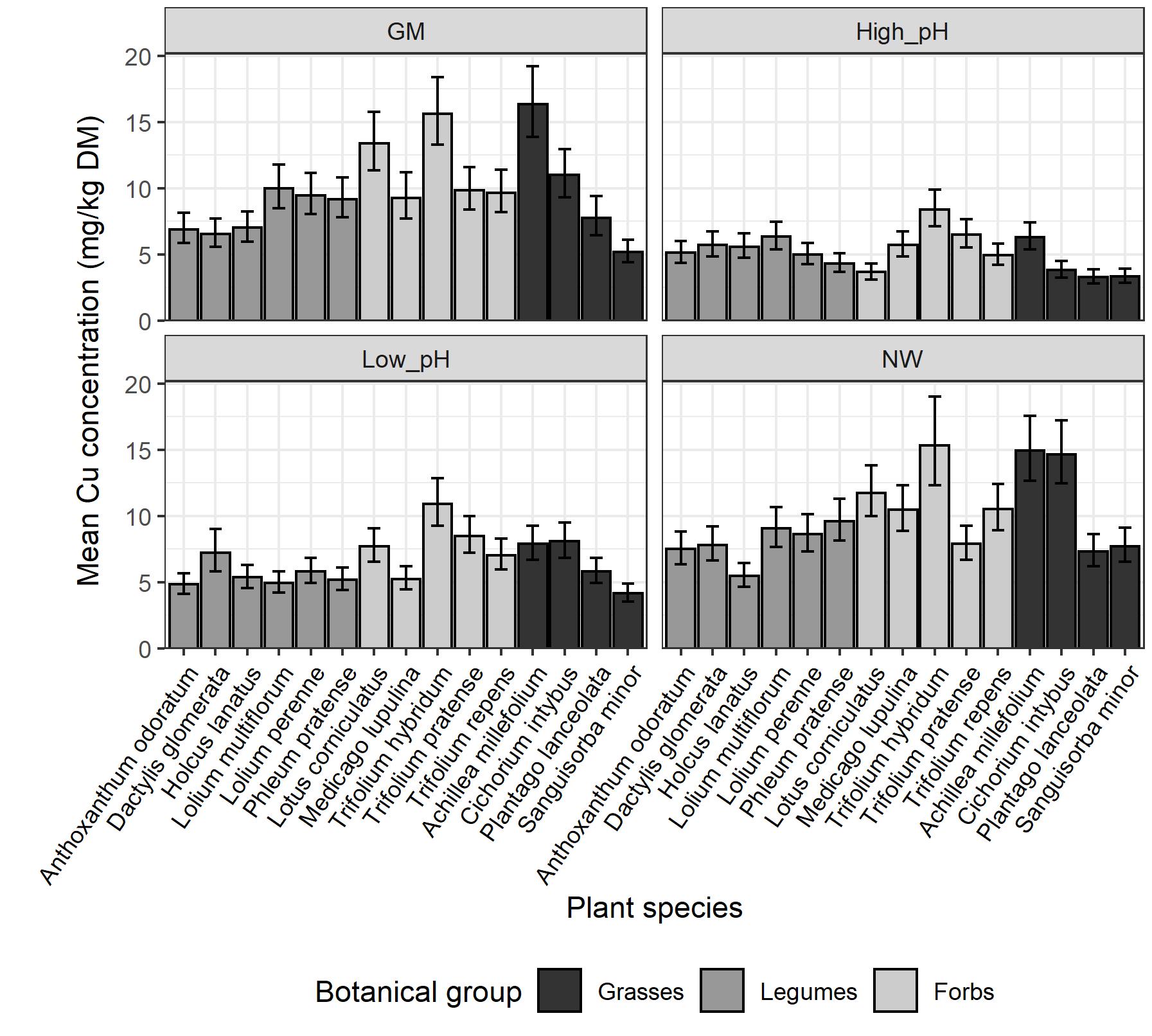

Supplement: S16 Fig — Error bars indicate the confidence interval of the back-transformed mean. (JPEG) [file pone.0277091.s016.jpeg]

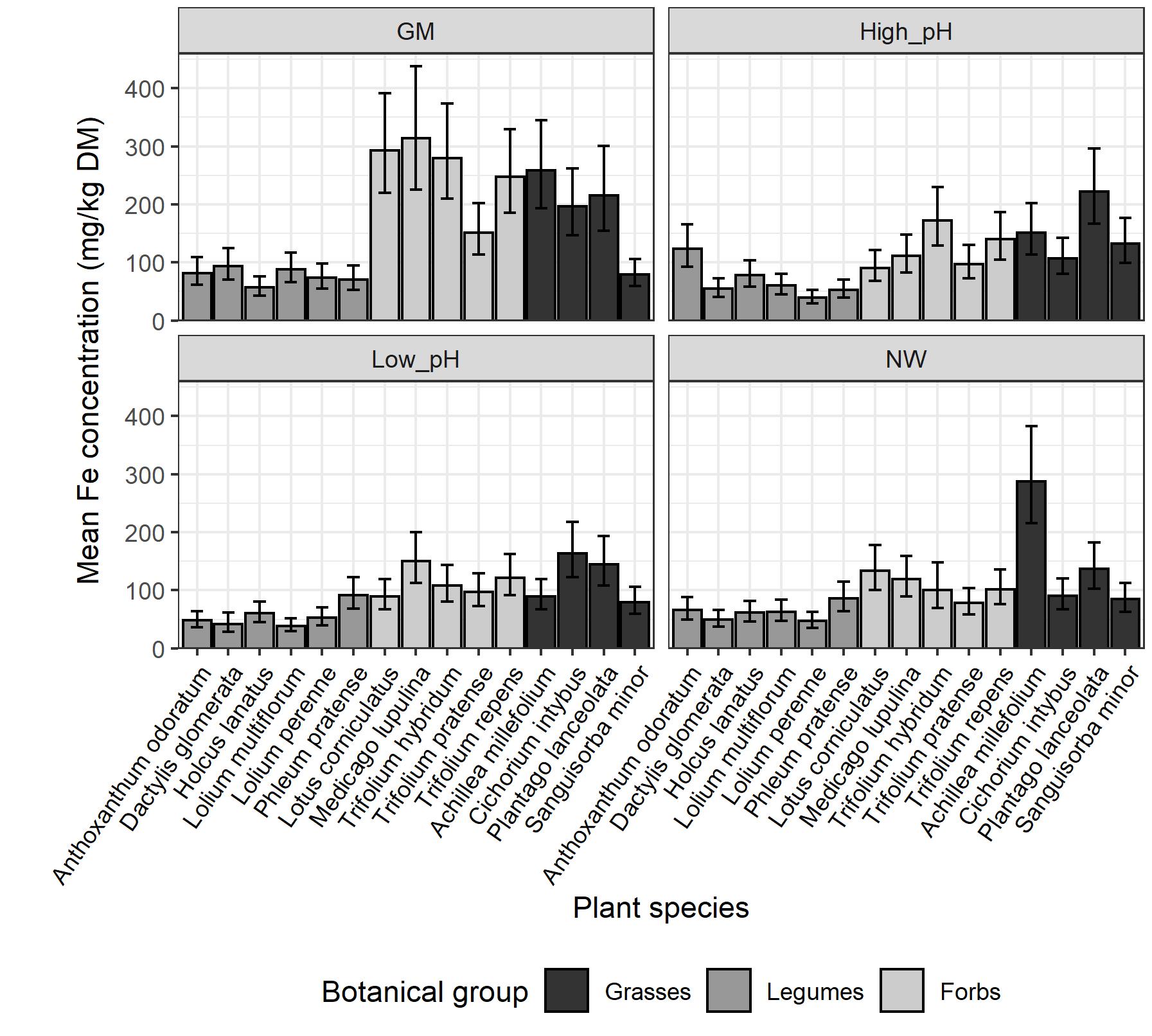

Supplement: S17 Fig — Error bars indicate the confidence interval of the back-transformed mean. (JPEG) [file pone.0277091.s017.jpeg]

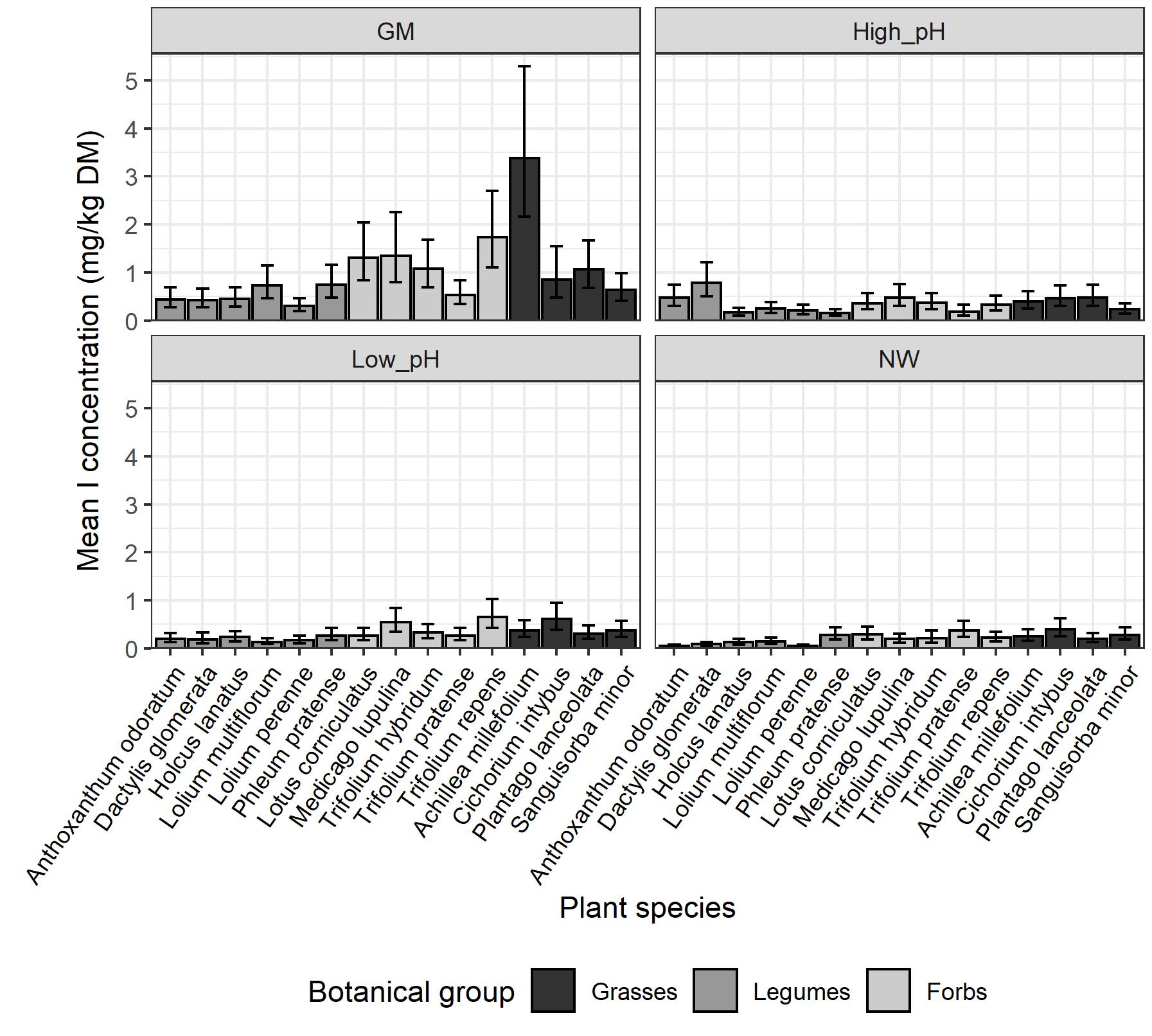

Supplement: S18 Fig — Error bars indicate the confidence interval of the back-transformed mean. (JPEG) [file pone.0277091.s018.jpeg]

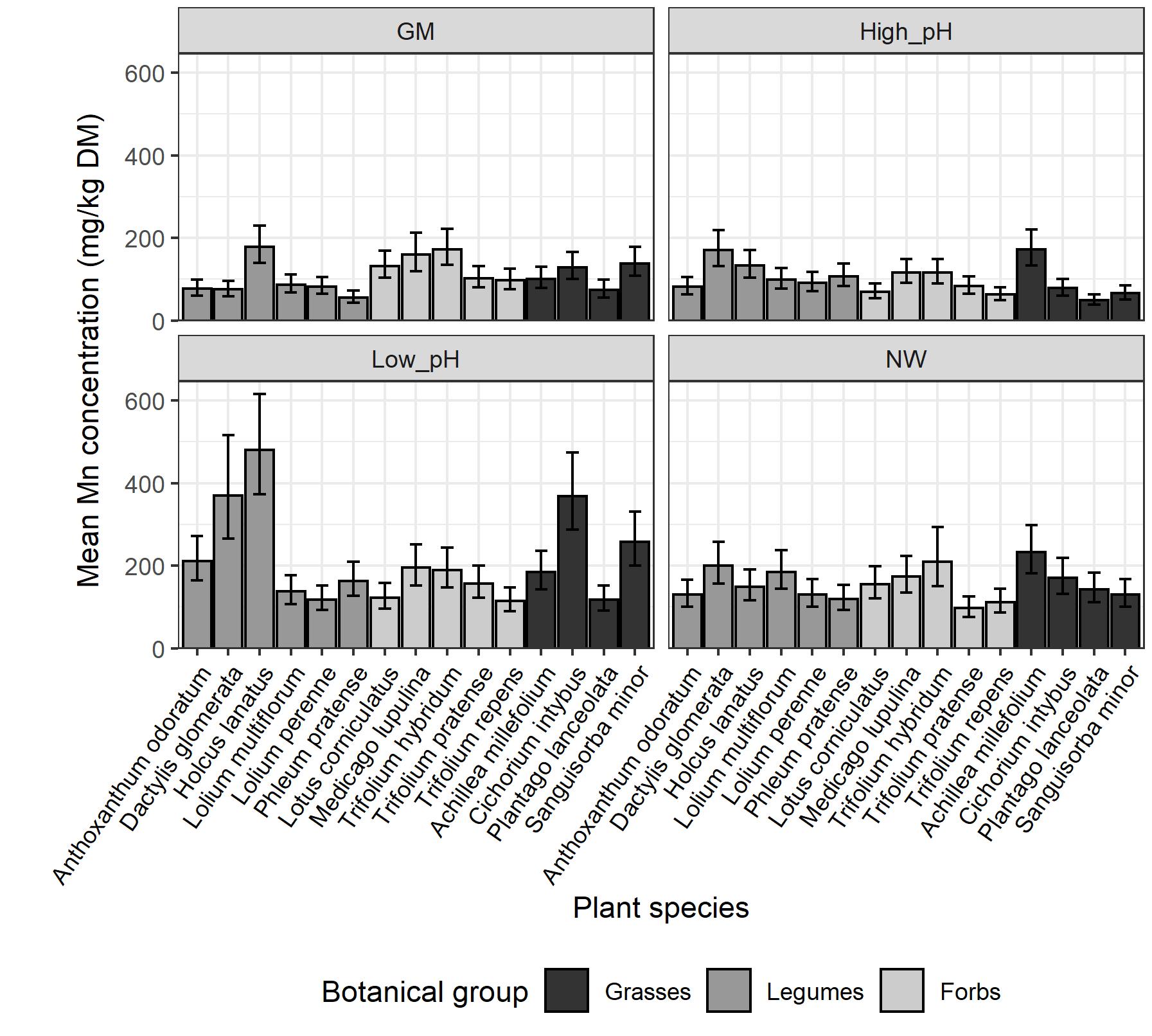

Supplement: S19 Fig — Error bars indicate the confidence interval of the back-transformed mean. (JPEG) [file pone.0277091.s019.jpeg]

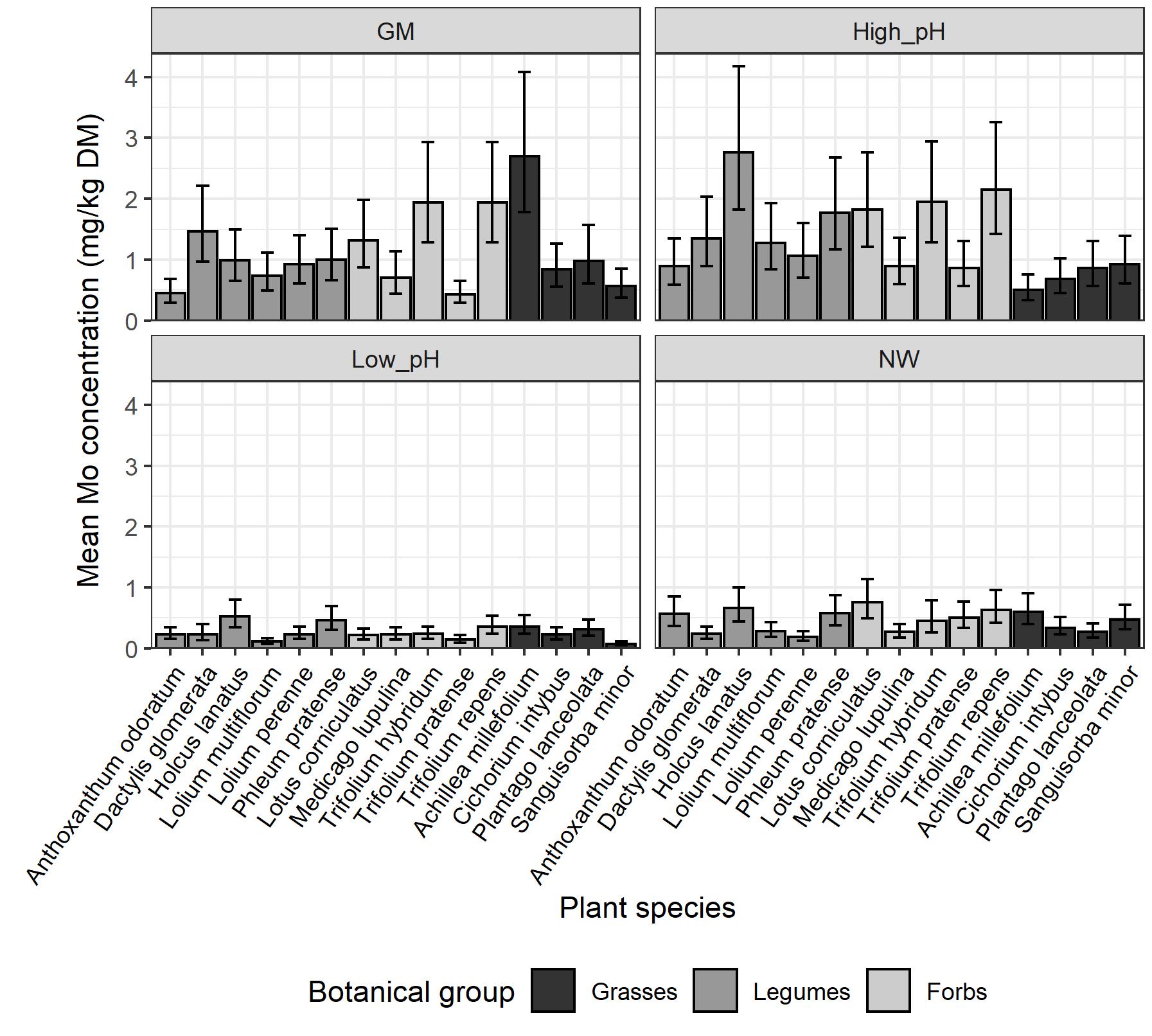

Supplement: S20 Fig — Error bars indicate the confidence interval of the back-transformed mean. (JPEG) [file pone.0277091.s020.jpeg]

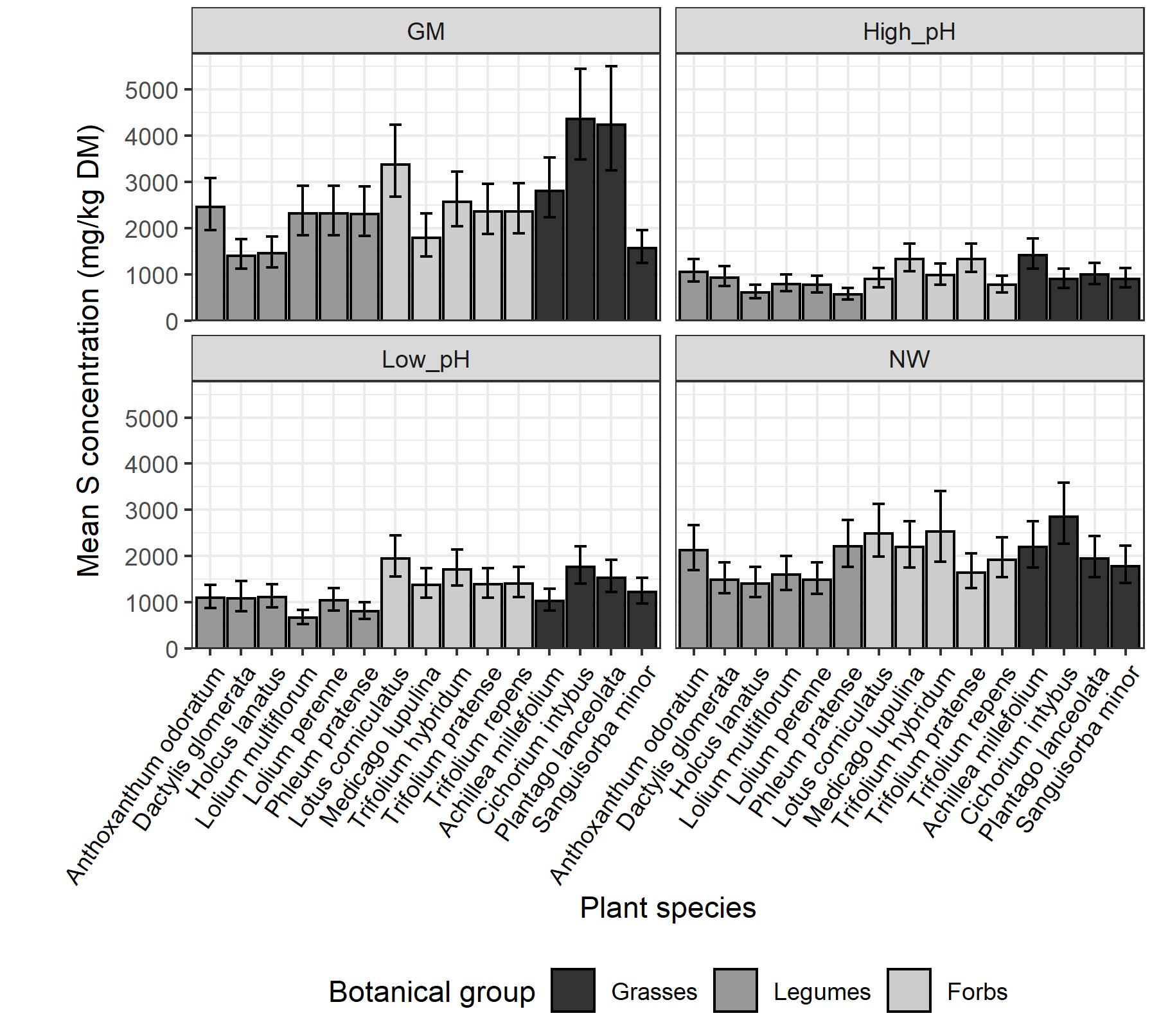

Supplement: S21 Fig — Error bars indicate the confidence interval of the back-transformed mean. (JPEG) [file pone.0277091.s021.jpeg]

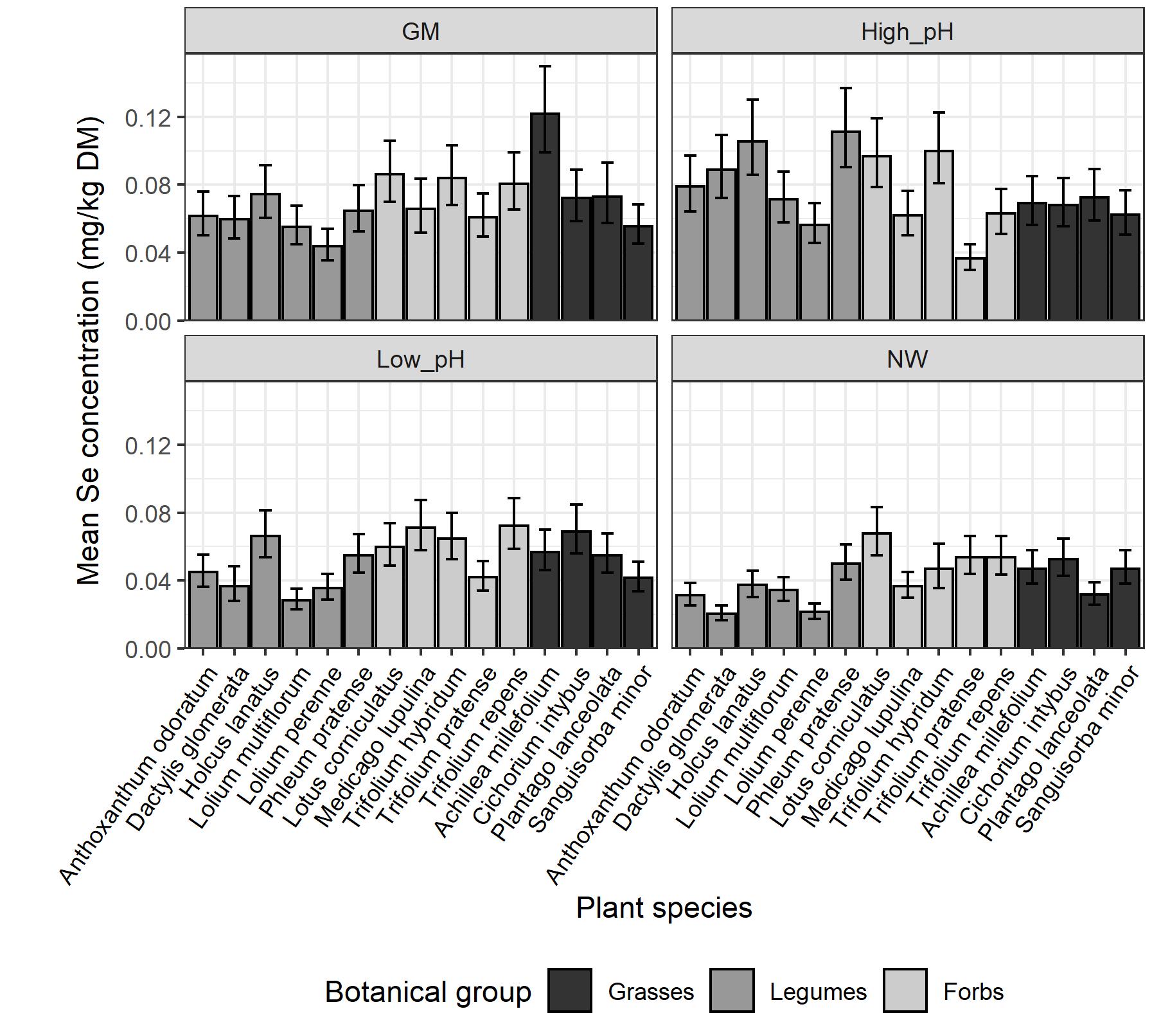

Supplement: S22 Fig — Error bars indicate the confidence interval of the back-transformed mean. (JPEG) [file pone.0277091.s022.jpeg]

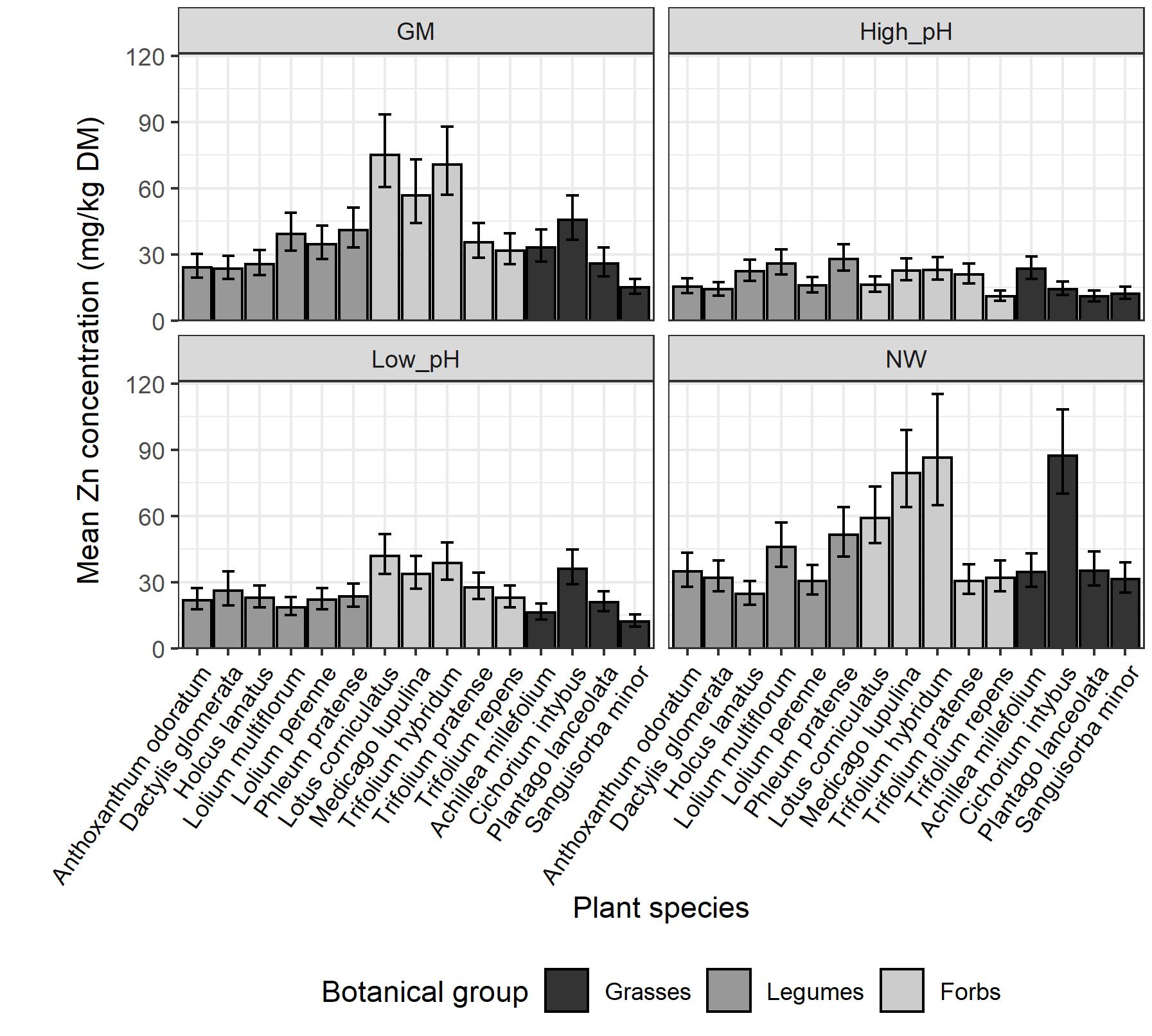

Supplement: S23 Fig — Error bars indicate the confidence interval of the back-transformed mean. (JPEG) [file pone.0277091.s023.jpeg]

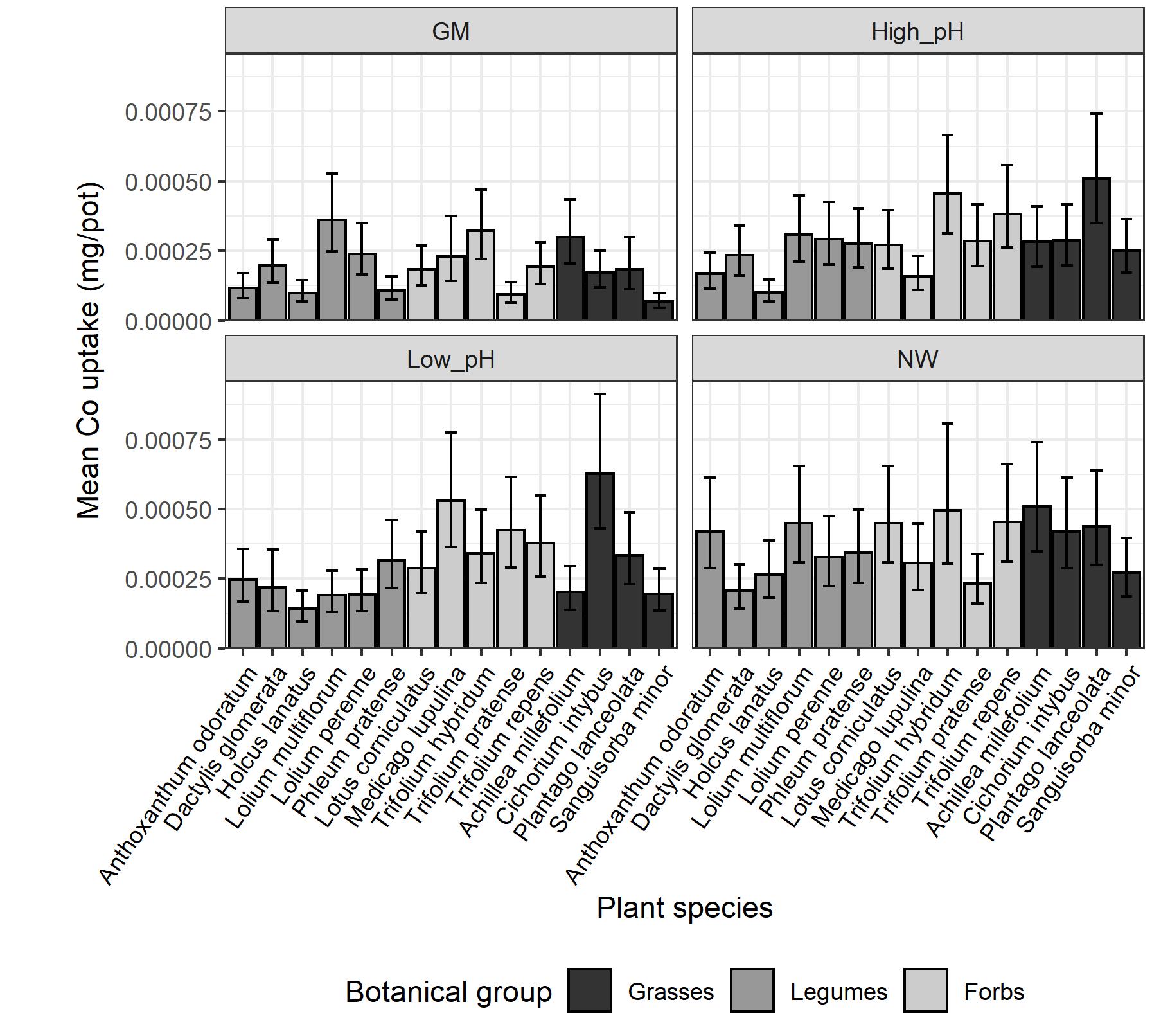

Supplement: S24 Fig — Error bars indicate the confidence interval of the back-transformed mean. (JPEG) [file pone.0277091.s024.jpeg]

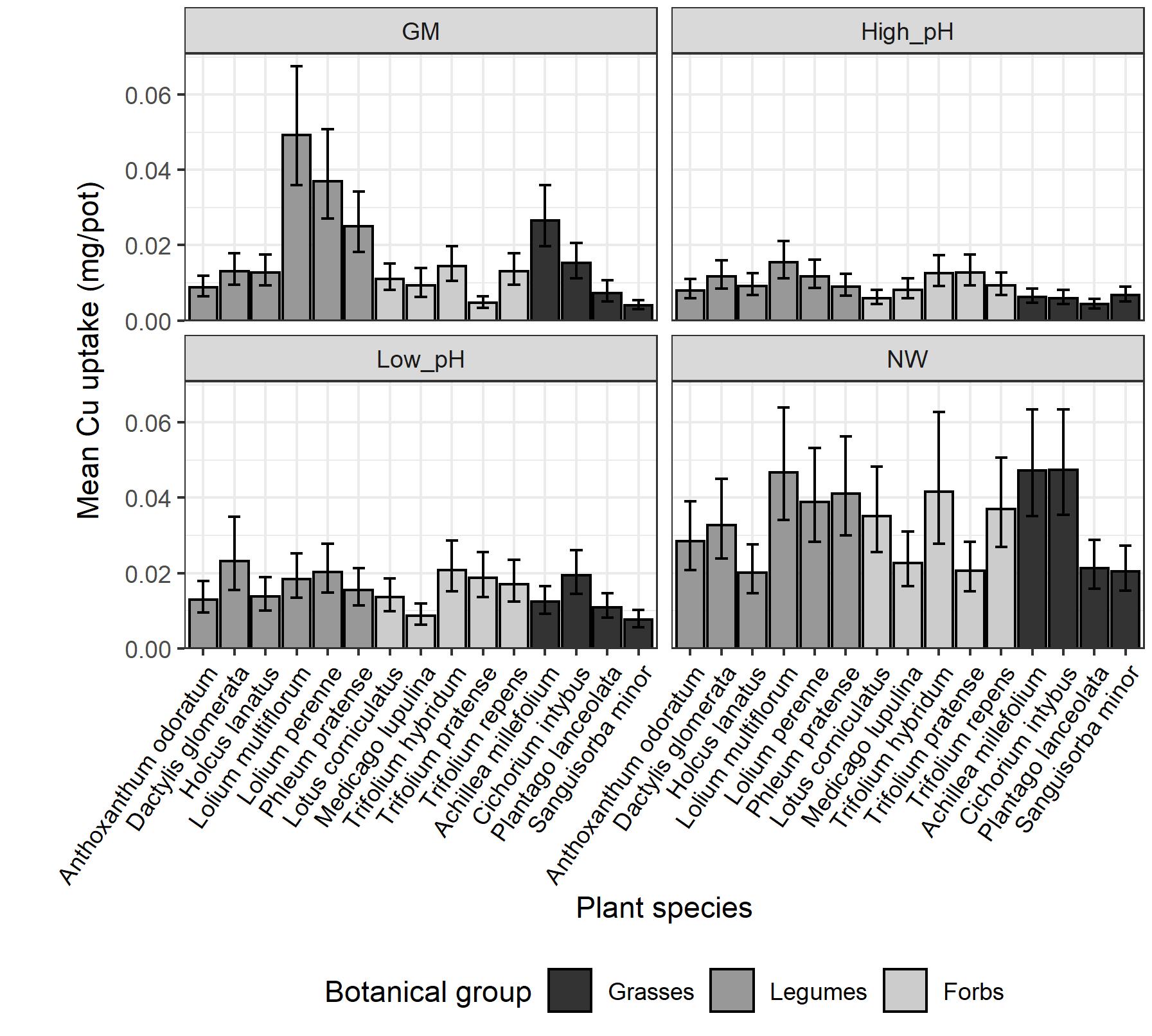

Supplement: S25 Fig — Error bars indicate the confidence interval of the back-transformed mean. (JPEG) [file pone.0277091.s025.jpeg]

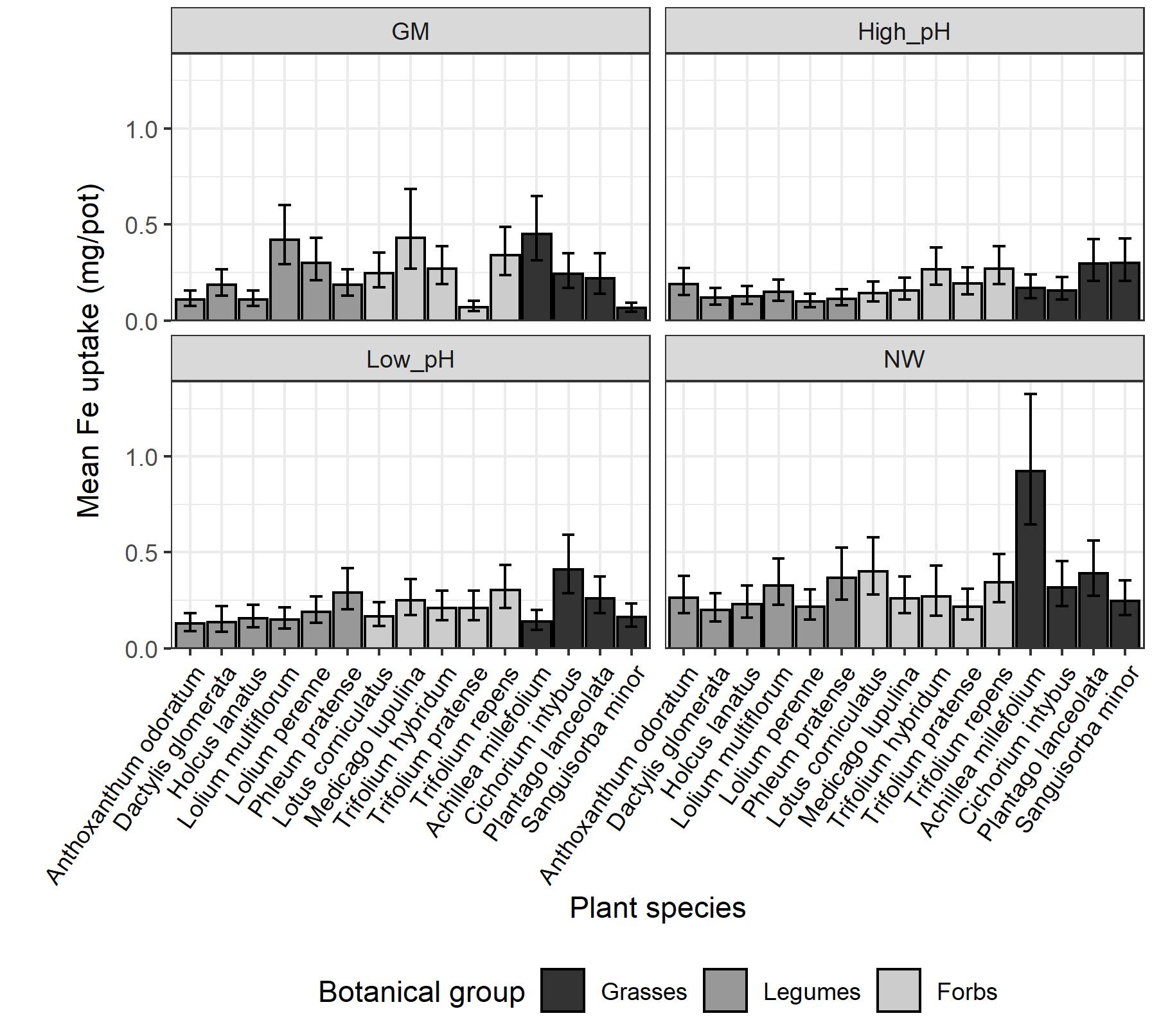

Supplement: S26 Fig — Error bars indicate the confidence interval of the back-transformed mean. (JPEG) [file pone.0277091.s026.jpeg]

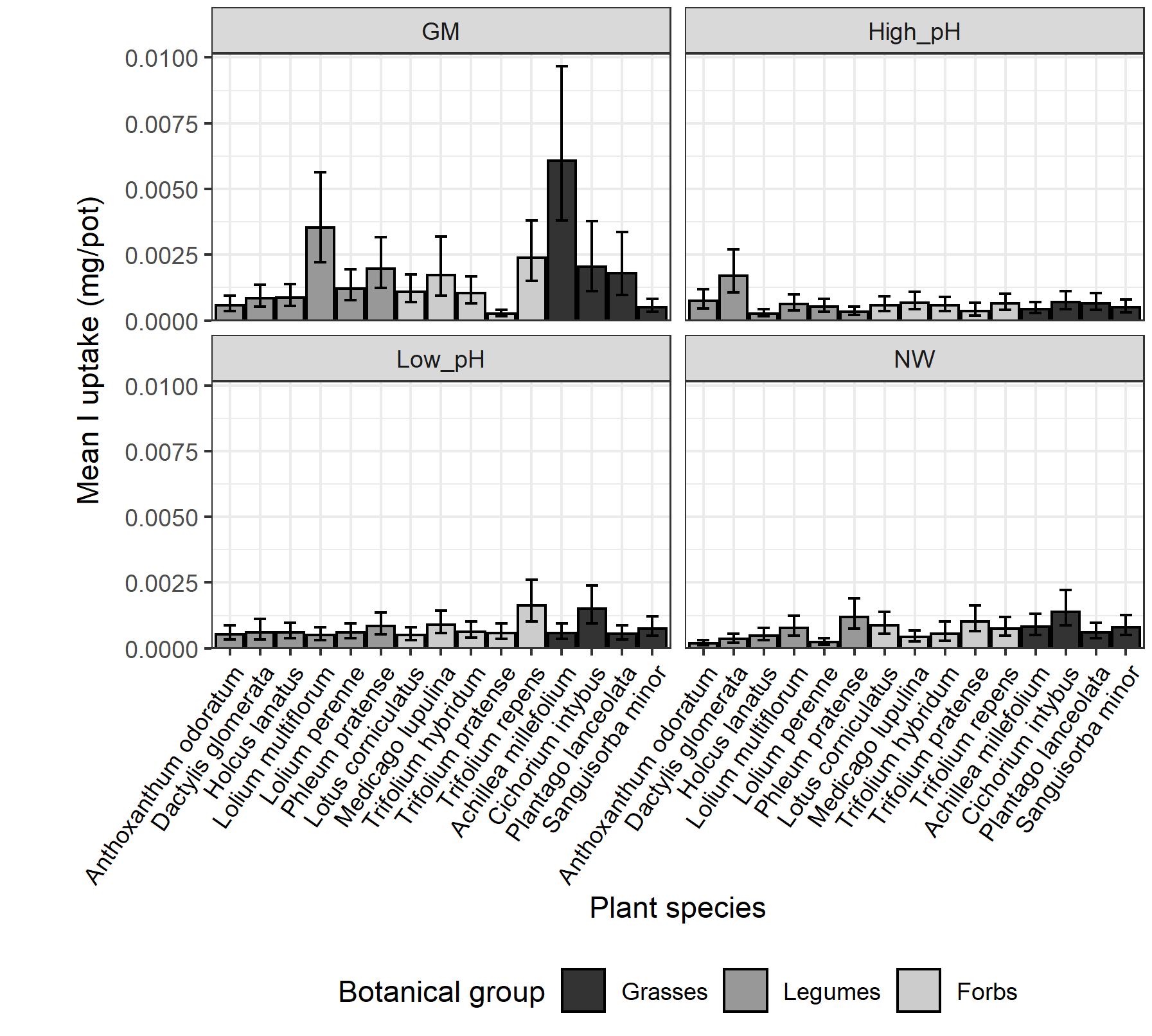

Supplement: S27 Fig — Error bars indicate the confidence interval of the back-transformed mean. (JPEG) [file pone.0277091.s027.jpeg]

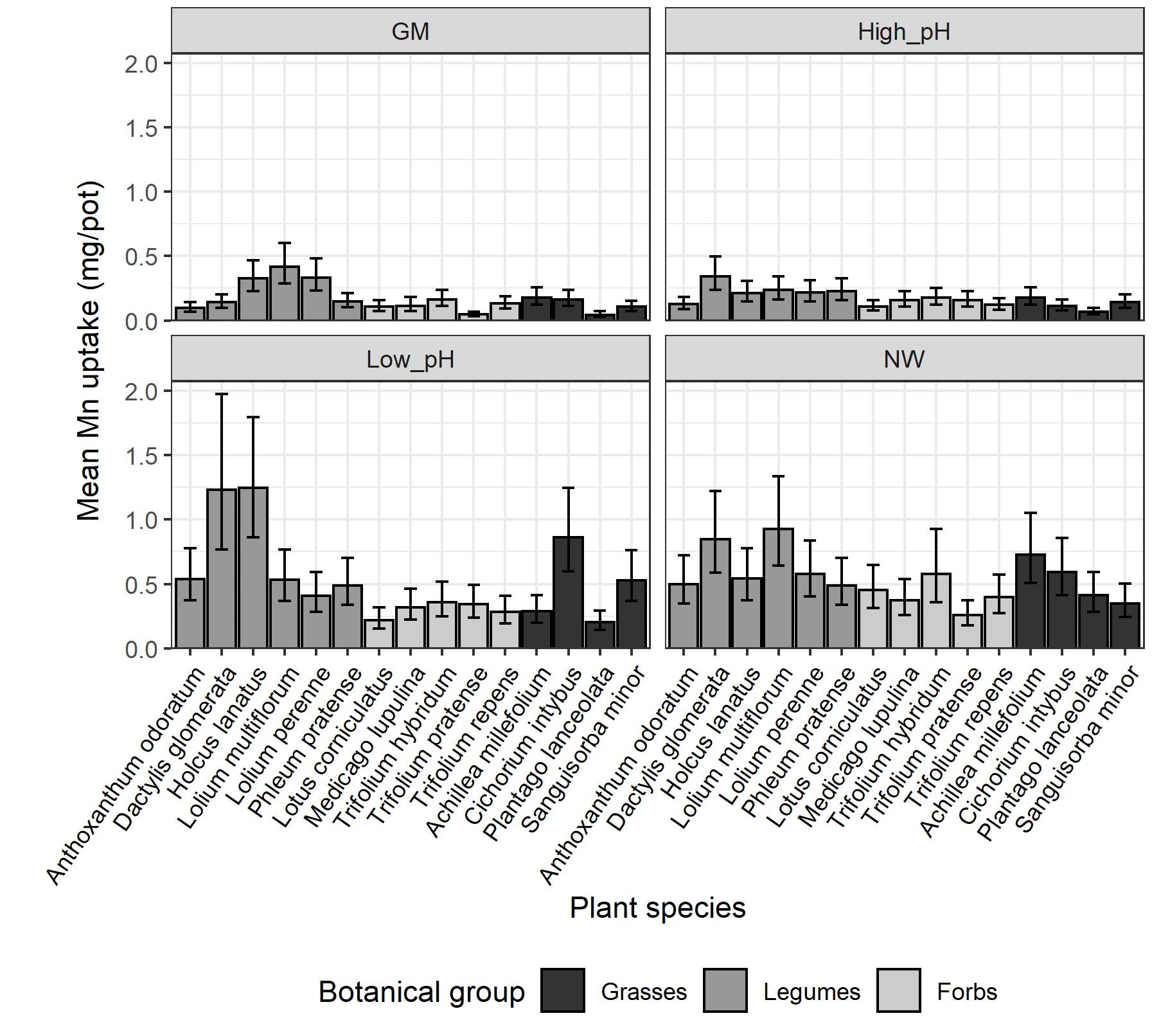

Supplement: S28 Fig — Error bars indicate the confidence interval of the back-transformed mean. (JPEG) [file pone.0277091.s028.jpeg]

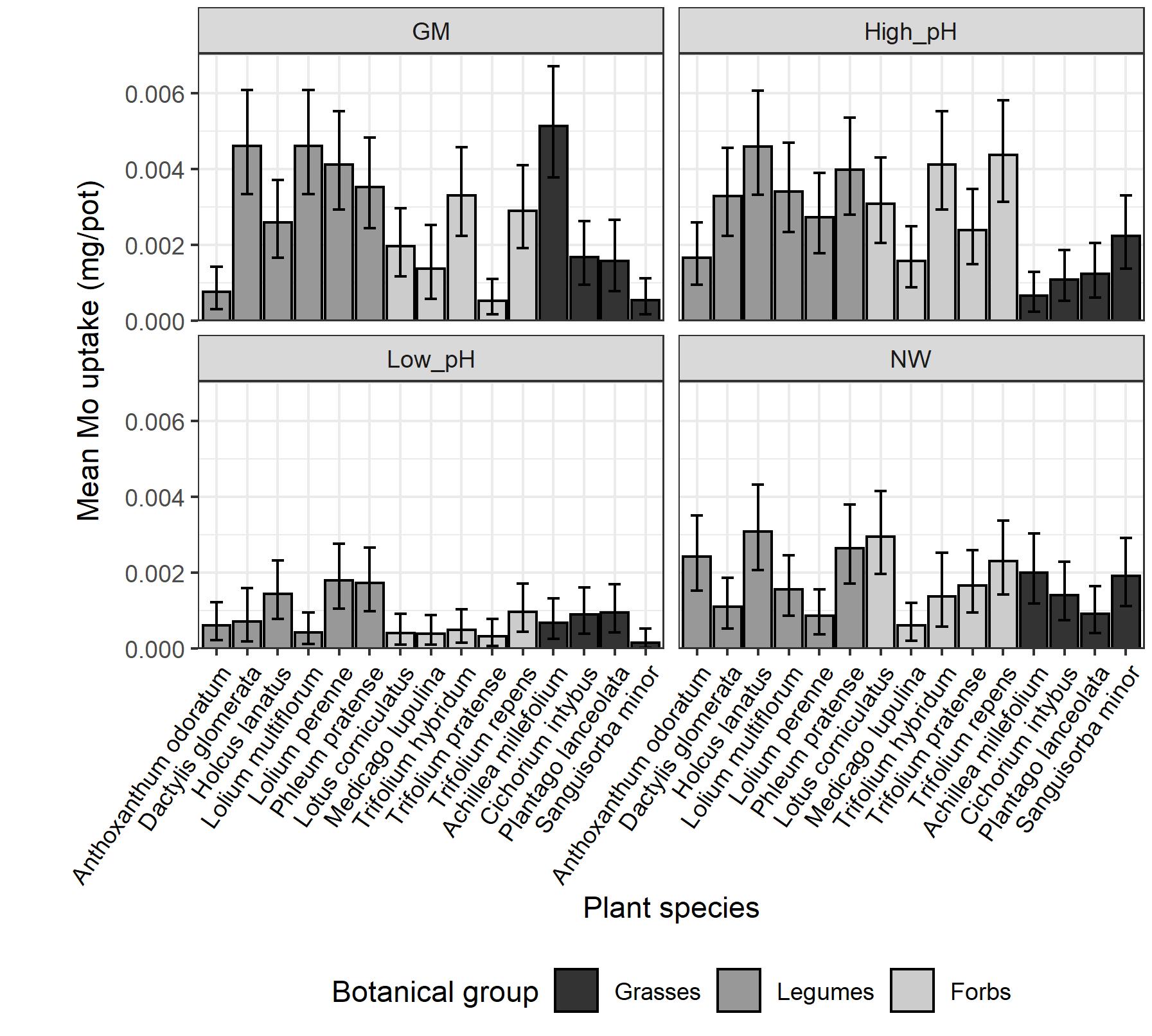

Supplement: S29 Fig — Error bars indicate the confidence interval of the back-transformed mean. (JPEG) [file pone.0277091.s029.jpeg]

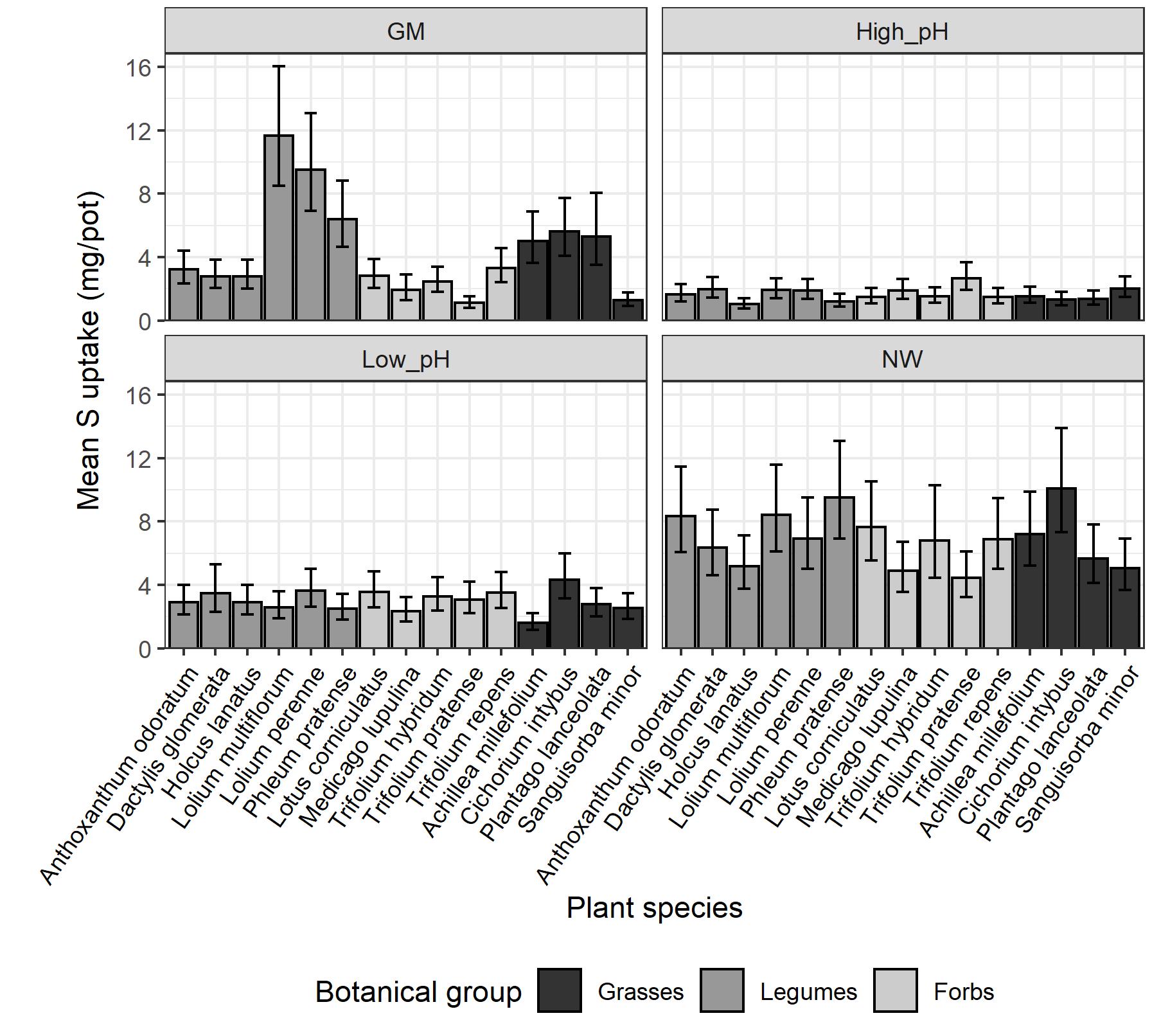

Supplement: S30 Fig — Error bars indicate the confidence interval of the back-transformed mean. (JPEG) [file pone.0277091.s030.jpeg]

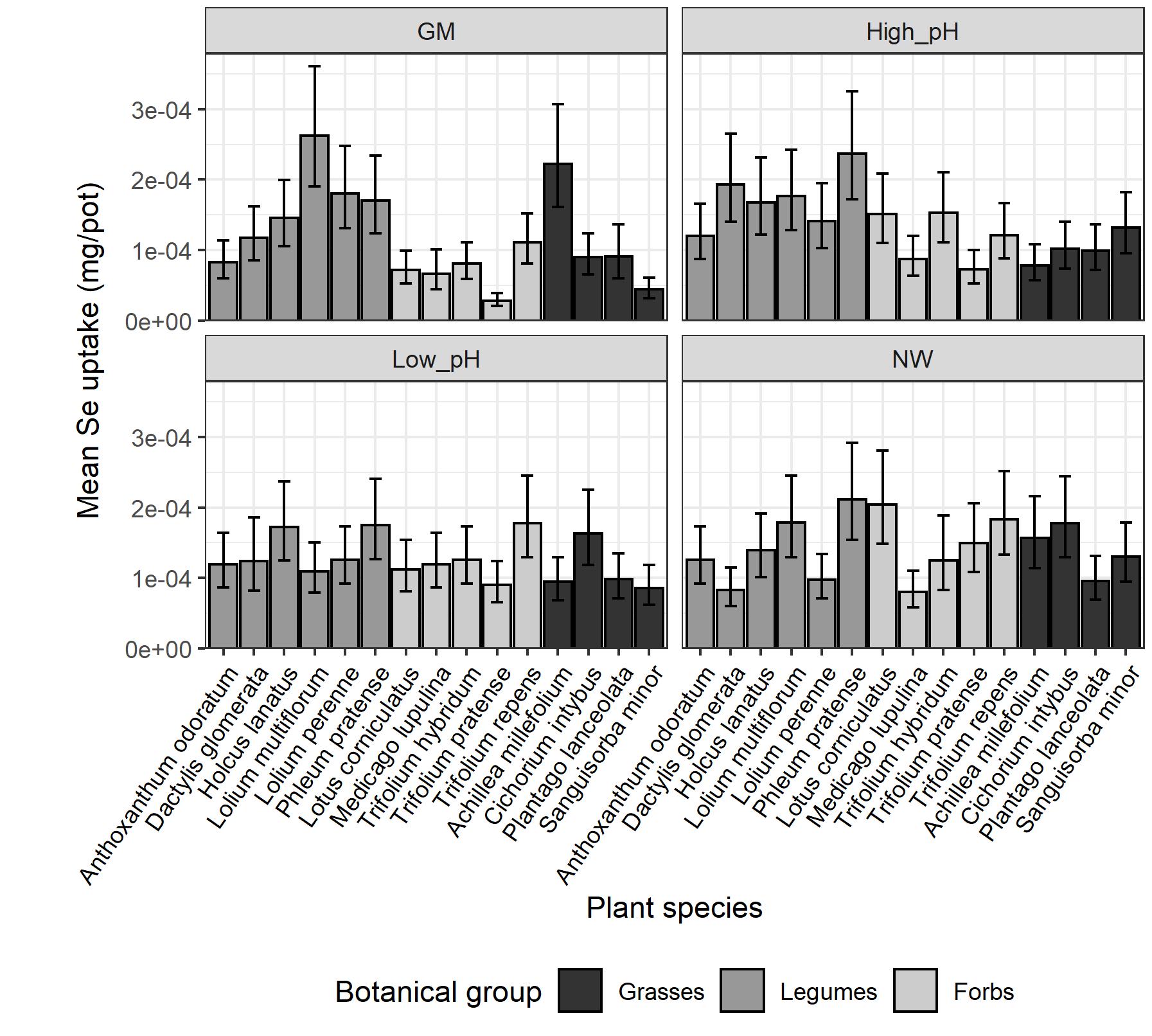

Supplement: S31 Fig — Error bars indicate the confidence interval of the back-transformed mean. (JPEG) [file pone.0277091.s031.jpeg]

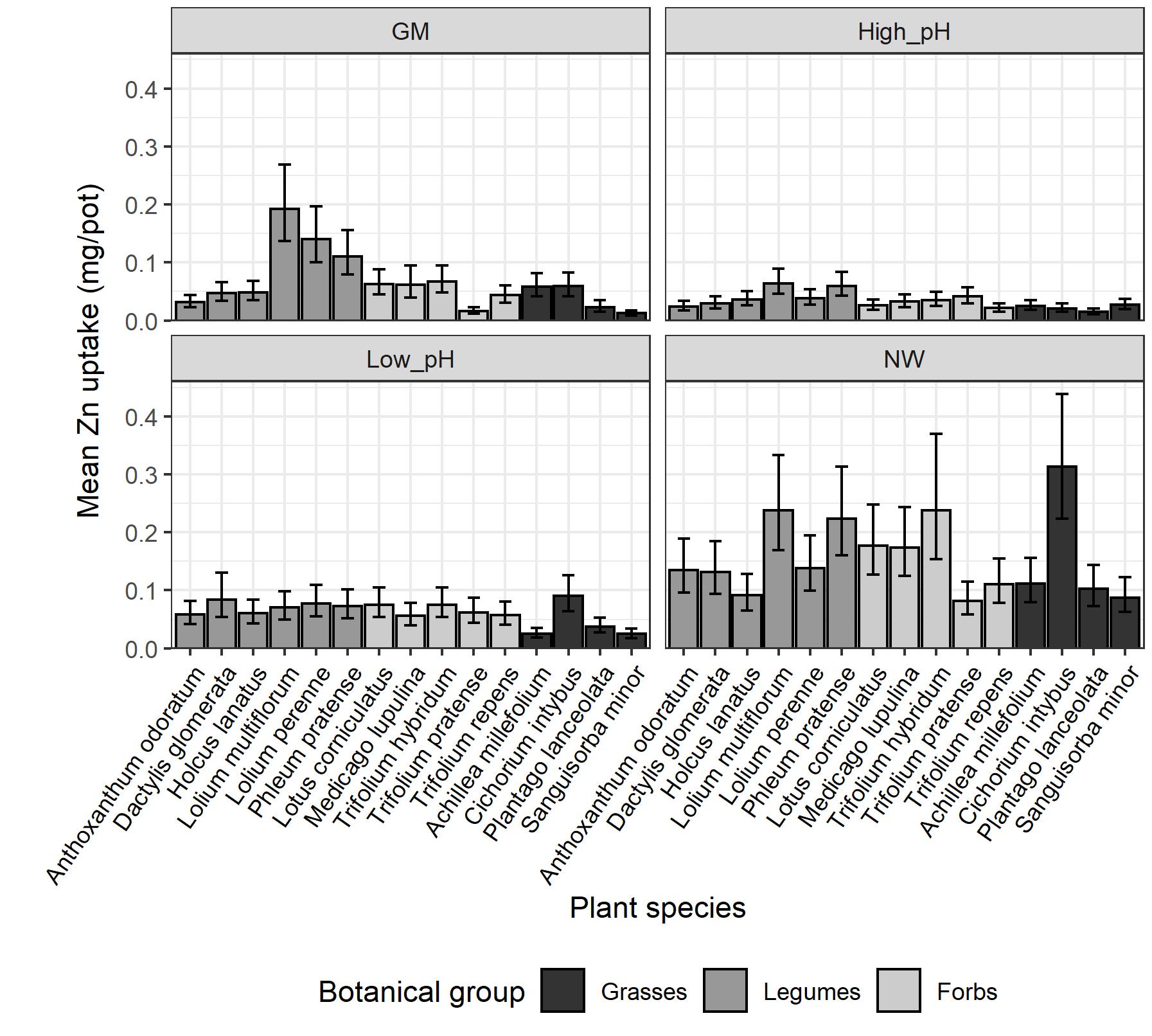

Supplement: S32 Fig — Error bars indicate the confidence interval of the back-transformed mean. (JPEG) [file pone.0277091.s032.jpeg]
